# Internal constraints and arrested relaxation in main-chain nematic elastomers

**DOI:** 10.1038/s41467-021-21036-3

**Published:** 2021-02-04

**Authors:** Takuya Ohzono, Kaoru Katoh, Hiroyuki Minamikawa, Mohand O. Saed, Eugene M. Terentjev

**Affiliations:** 1 Research Institute for Electronics and Photonics, National Institute of Advanced Industrial Science and Technology (AIST), Tsukuba, Japan; 2 Biomedical Research Institute, AIST, Tsukuba, Japan; 3 Research Institute for Sustainable Chemistry, AIST, Tsukuba, Japan; 4 Cavendish Laboratory, University of Cambridge, Cambridge, UK

**Keywords:** Liquid crystals, Polymers, Structure of solids and liquids

## Abstract

Nematic liquid crystal elastomers (N-LCE) exhibit intriguing mechanical properties, such as reversible actuation and soft elasticity, which manifests as a wide plateau of low nearly-constant stress upon stretching. N-LCE also have a characteristically slow stress relaxation, which sometimes prevents their shape recovery. To understand how the inherent nematic order retards and arrests the equilibration, here we examine hysteretic stress-strain characteristics in a series of specifically designed main-chain N-LCE, investigating both macroscopic mechanical properties and the microscopic nematic director distribution under applied strains. The hysteretic features are attributed to the dynamics of thermodynamically unfavoured hairpins, the sharp folds on anisotropic polymer strands, the creation and transition of which are restricted by the nematic order. These findings provide a new avenue for tuning the hysteretic nature of N-LCE at both macro- and microscopic levels via different designs of polymer networks, toward materials with highly nonlinear mechanical properties and shape-memory applications.

## Abstract (web summary)

Nematic liquid crystal elastomers (N-LCE) have a slow relaxation, which can prevent their shape recovery. Here, the authors examine mechanical hysteresis in a series of main-chain N-LCE to understand how the inherent nematic order retards and arrests the equilibration.

## Introduction

Liquid crystal elastomers^1^ (LCE) exhibit many remarkable properties that offer attractive applications, including soft actuators^2–5^, shape morphing^6,7^, shape memory^8–11^, mechanical damping^12,13^ dynamic adhesion^14,15^ and friction^16^. These intriguing properties stem from the coupling between the LC order^17^ and the thermally mobile polymer network^18^. Orientational order imposes strong anisotropic constraints to the network structure. The striking feature, well studied in the equilibrium regime, is the soft elasticity, which manifests as a wide plateau of low nearly constant stress upon increasing strain, and is caused by internal rotation of the local director axis^19–21^, absorbing applied strain without elastic energy cost^1,22–25^.

In contrast, the understanding of non-equilibrium features, such as slow stress relaxation, hysteretic behaviour and their relationship to the soft elastic response and associated nematic domain evolution^20,26,27^, has so far been limited. Some of these phenomena in isolation have been explored for nematic (N) LCE^1,28–30^. In smectic LCE^31^, it has been long established that the coupling between crosslinks and the layer positions^32,33^ leads to strong constraints preventing those crosslinks moving between the layer planes, resulting in the pronounced hysteretic shape-memory effects^8–11^. In contrast, in N-LCE with only the orientational nematic interactions, the origin of the very slow relaxation, especially of the arrested states found in the main-chain N-LCE^28–30^, remains unclear. Here we investigate the hysteretic properties of a series of specially designed N-LCE, manifesting in both macroscopic stress–strain responses and microscopic nematic domain transformation. We use the concept of potential energy landscape (PEL)^34^, familiar in phenomenological description of the glass transition, the arrest phenomena^35,36^ and protein folding^37^. As the internal constraints promoting the retardation and arrested relaxation observed in a series of N-LCE, we propose the effect of hairpin dynamics^18,38^ to be responsible, with energetically costly chain hairpins transiently defying the local nematic order and inherently accompanying the internal director rotation^19–21^ in the main-chain N-LCE.

## Results

### Macroscopic hysteresis on mechanical responses

**Fig. 1:**
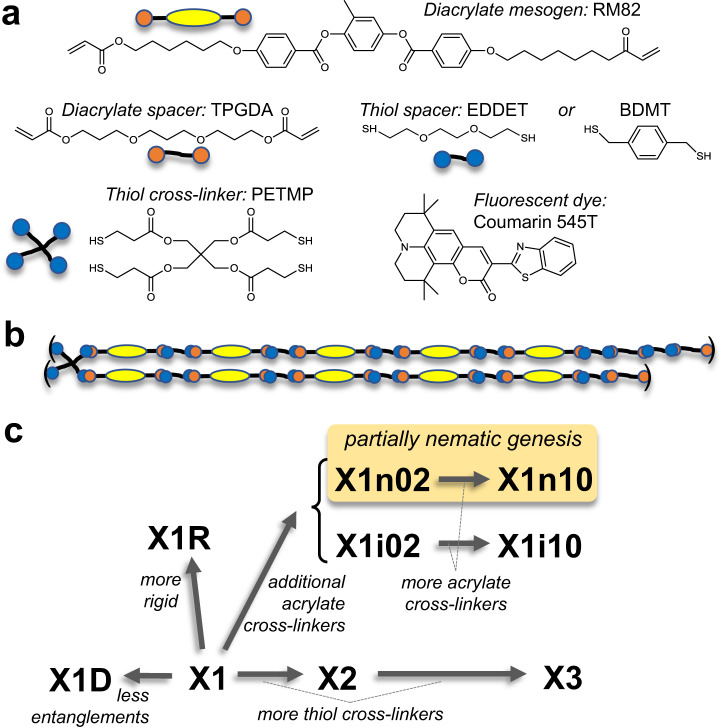
Main-chain nematic liquid crystal elastomers (LCE). **a** Chemical formulae of the components. **b** Schematic of a nominal polymer unit for X1. **c** Schematics of present nine LCE showing main differences from X1 (see Methods, Supplementary Fig. 1, and Supplementary Table 1 for details). The thiol cross-linker concentration in X2 and X3 were increased from that in X1. X1D was cross-linked with solvent and de-swollen diminishing network entanglements compared to X1. In X1R, the rigid thiol spacer BDMT was used instead of EDDET in X1. In X1i02, X1i10, X1n02 and X1n10, the acrylates excess to thiols are photo-polymerised after the first cross-linking reaction, producing additional crosslinks. X1i02 and X1i10 are photo-polymerised at 75 °C (isotropic). Meanwhile, X1n02 and X1n10 are photo-polymerised at 20 °C (nematic) to produce partial nematic genesis^24^ LCE. The last numbers, “02” and “10”, indicate the excess% amount of the acrylates.

Nine room-temperature N-LCE were prepared with slightly different procedures and/or monomers (Methods, Fig. 1, Table 1, Supplementary Figs. 1–2, Supplementary Table 1) to discuss the origin of the hysteretic behaviour. The effective crosslink density, *n*_XD_, in X1 was doubled or quadrupled in X2 or X3, respectively. *n*_XD_ in X1D is lower than in X1 due to the fewer network entanglements. In X1R, strands are more rigid than X1. In X1i– or X1n–, the acrylate units excess to thiols were introduced to create crosslinks between them in addition to thiol crosslinkers and were subsequently photo-polymerised at isotropic or nematic temperature, respectively. Numbers, 02 and 10, indicate the acrylate excess% amount, reflecting the associated increase in *n*_XD_.

**Table 1:** Properties of main-chain nematic LCEs. For details, see Methods, Supplementary Figs. 1–2, Supplementary Table 1 and Figs. 2–3.

			X1D	X1	X1R	X2	X3	X1i02	X1i10	X1n02	X1n10
Nominal network properties	*n*_*XD*_ [nm^−3^]		0.059	0.059	0.059	0.116	0.226	0.068	0.103	0.068	0.103
	*L*_*XD*_ [nm]		2.58	2.58	2.56	2.05	1.64	2.45	2.13	2.45	2.13
	*L*_CON_ [nm]		31.97	31.97	30.67	16.16	8.25	28.81	21.22	28.81	21.22
	*L*_CON_/*L*_*X*D_ [-]		12.4	12.4	12.0	7.9	5.0	11.8	9.9	11.8	9.9
Thermal properties	*T*_NI_ [°C]		43 ± 2	41 ± 1	51 ± 3	42 ± 2	49 ± 3	45 ± 2	48 ± 2	54 ± 3	67 ± 3
	*T*_g_^*^ [°C]		−24	−26	−1	−18	−8	−27	−21	−21	−19
Mechanical properties	*e* at break [-]		3.4	2.0	1.7	1.7	1.0	1.7	0.8	2.1	2.8
	upper *e* of soft range [-]		0.50	0.40	0.35	0.22	− no soft range	0.23	− no soft range	0.50	0.5-0.7
	*σ*_*c*_ [kPa]		less than 0.3	less than 0.3	less than 0.3	less than 0.3	−	less than 0.3	−	27 ± 5	100~170 soft range with slope
	viscoelastic parameters of *σ*(*ė*) at *e* = *e*_*s*_ fitted to *σ* ~ *σ*_r_ + *νė*^*m*^	*e*_*s*_ [-]	0.3	0.3	0.3	0.2	0.1	0.2	0.1	0.3	0.3
		*σ*_r_ [MPa]	0	0	0	0	0.052	0	0.01	0.023	0.123
		*ν* [MPa s^−*m*^]	0.05	0.33	0.94	0.38	0.36	0.24	0.14	0.12	0.27
		*m* [-]	0.40	0.43	0.40	0.43	0.41	0.49	0.48	0.27	0.28
Macroscopic hysteresis	*e*_*r*_ [-] at *t* = 10^3^−10^6^ s		0.53	0.45	0.42	0.25	0 recovery	0.25	0 recovery	0 recovery	0 recovery
Microscopic hysteresis	Recovery of director patterns at *t* ~10^3^ s		No recovery	No recovery	No recovery	No recovery	No recovery	No recovery	No recovery	recovery	recovery

^a^*n*_*XD*_, the nominal number density of the crosslinks per unit volume. *L*_*XD*_, the nominal distance between cross-links. *L*_CON_, the contour length of a nominal strand between nearest neighbouring crosslinks. Note that X1D has less network entanglements, and thus, the lowest effective cross-link density.

^b^*T*_g_*, Upper limit of glass transition temperature.

^c^Macroscopic and microscopic hysteresis indicate the abilities to recover initial states after a cycle of straining and release.

**Fig. 2:**
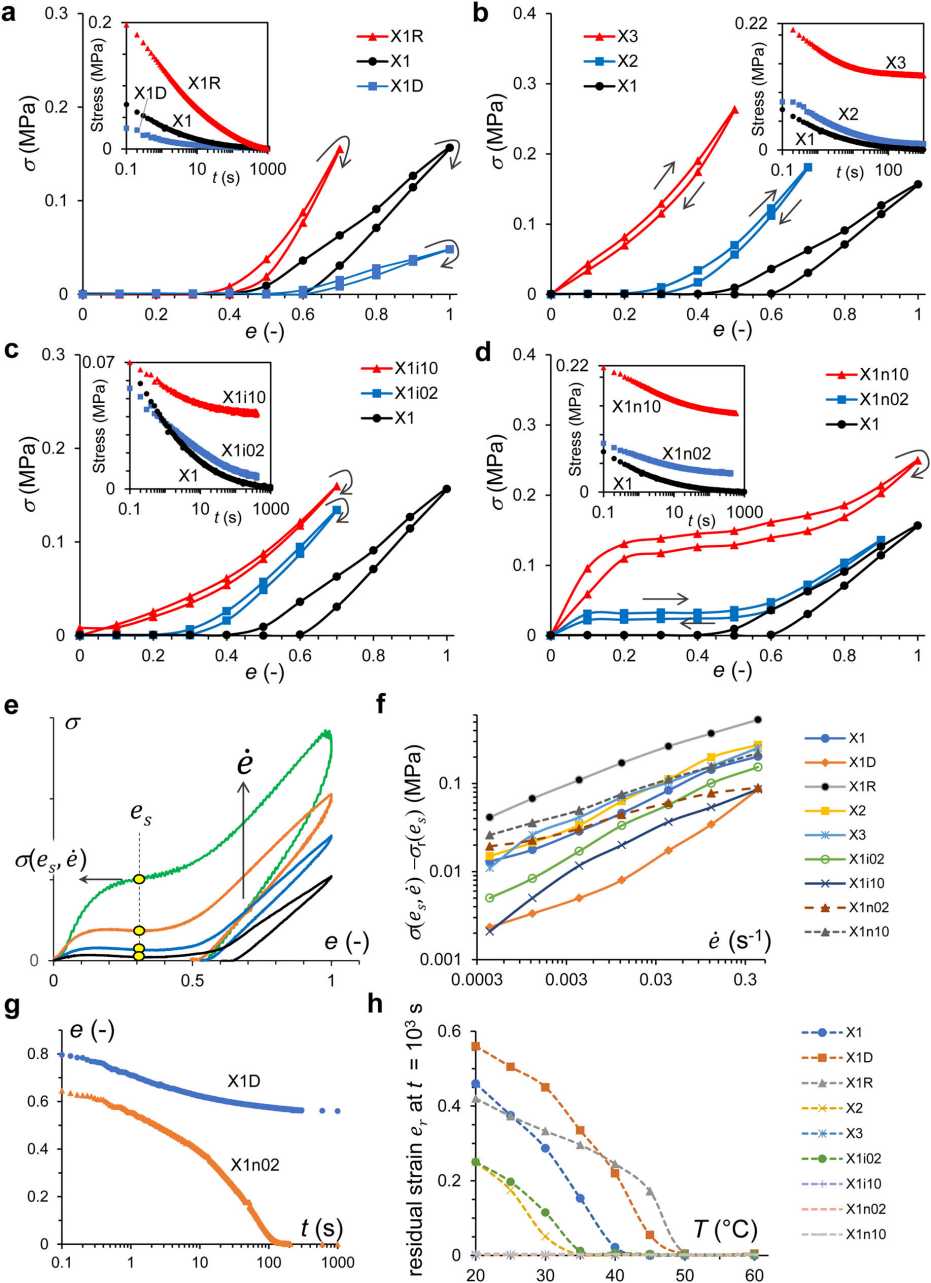
Mechanical properties. **a**–**d** Equilibrated stress, *σ*, vs. strain, *e* = (*L* − *L*_0_)/*L*_0_, taken after strain increment of 0.1 followed by equilibration (Supplementary Fig. S3) for 10^3^ s (X1D, X1R, X1, X2, X3) or 400 s (X1i02, X1i10, X1n02, X1n10) at *T* = 20 °C (nematic phase). The plot of X1 is shown in all panels for comparison. Note that some of the soft plateau stress *σ*_*c*_ are close to zero (Table 1). (Insets) Typical stress-relaxation curves at strain of *e*_*up*_ = 0.3 upon increasing strain. **e**–**f** $\dot e$-dependence of *σ* at a certain strain *e*_*s*_ at soft elastic range at *T* = 20 °C. **e** Typical strain rate, $\dot e$–dependent *σ–e* curves. **f** Plots of $\log \left( {\dot e} \right) - \log \left( {\sigma - \sigma _{{{\mathrm{r}}}}} \right)$. Values of *e*_*s*_ and parameters fitted to $\sigma = \sigma _{{{\mathrm{r}}}} + \nu \dot e^m$ were shown in Table 1. **g** Representative strain recovery *e*(*t*) of X1D (hysteretic) and X1n02 (reversible) at *T* = 20 °C. **h** The residual strain, *e*_*r*_, after relaxation at various *T*. The spontaneous strain rates have already become very low; at least $\dot e$ < 10^−6^ s^−1^, after equilibration of 10^3^ s. While X3, X1i10, X1n02 and X1n10 show full recovery of the original strain at any temperature, others show residual stains, *e*_*r*_ ≠ 0, in the nematic phase.

Naturally non-aligned, polydomain LCE with relatively low *n*_XD_ show stress–strain curves with a typical stress plateau reflecting the elastic softness on nematic director alignment, often called the polydomain–monodomain transition^20,27,39^ (Fig. 2a–d, Supplementary Fig. 3). While the plateau stress apparently shows non-zero values upon constant strain rate, $\dot e$ (Supplementary Figs. 4–6), it relaxes to zero, being less than the stress resolution of ~0.3 kPa (Table 1), in X1D, X1, X1R, X2 and X1i02 after equilibration of 0.4–1 × 10^3^ s, which is referred to as *σ*_*c*_. Such a low plateau stress, and the corresponding strain threshold of the polydomain-monodomain transition, are expected features of the isotropic-genesis N-LCE. After releasing the external strain (and load) applied to these LCE, the final states show a characteristic residual strain, *e*_*r*_, which roughly corresponds to the final data point in the stretching–releasing cycle in Fig. 2a–d, i.e. the upper limit of the soft plateau (Table 1). Since these arrested states are stable at least over months of time, strain relaxation is almost frozen on these LCE. In contrast, X3, X1i10, X1n02 and X1n10 recover the initial shape soon after releasing strain. In mechanically fragile X3 and X1i10 with higher *n*_XD_, the soft plateau range vanishes almost completely. In contrast, the partially nematic-genesis X1n02 and X1n10 show the soft plateau extended almost as much as in X1 (to strain of about 50%) and with non-zero *σ*_*c*_, which is the case typically reported as the reversible stress–strain response of N-LCE^30^ referred to as the “semi-soft elasticity” regime^1,24^, also related to the elastic energy stored upon the domain wall localisation^27^.

The rate-dependent $\sigma \left( {e_s,\dot e} \right)$ (Fig. 2e, f, Supplementary Fig. 7) characterises the relaxation process at a specific strain *e*_*s*_, which is chosen within the soft elastic range if it exists (Table 1). The power index *m*, when $\sigma \left( {e_s,\dot e} \right)$ is fitted to a model equation for power-law fluids, $\sigma \left( {e_s,\dot e} \right) = \sigma _r\left( {e_s} \right) + \nu \dot e^m$, where *σ*_r_(*e*_*s*_) is the reference stress at *ν* = 0 and *e* = *e*_*s*_, may measure how fast the viscous relaxation proceeds. The smaller exponent *m* suggests the faster relaxation, i.e. more elastic system, which is found in X1n–with the *m* ~0.27–0.28, comparing to others with *m* ~0.40–0.49. This indicates the difference emerging from the genesis of LCE^24^, which is the phase during crosslinking (Fig. 1c). In LCE showing the soft elastic range, *σ*_r_ roughly corresponds to the plateau *σ*_*c*_ (Table 1).

Typical shape-recovery processes *e*(*t*) after a sudden load release from a certain stretched state are shown in Fig. 2g for non- and full-recovery cases in the nematic phase (Supplementary Fig. 8 for others). In this experiment, the internal elastic stress alone drives the recovery, so it is not surprising that once the region of soft plateau is reached the recovery stops. Temperature-dependent residual strains, *e*_*r*_(*T*), after 20 min equilibration are plotted in Fig. 2h. Even in LCE with *e*_*r*_ ≠ 0 at 20 °C, *e*_*r*_ decreases with *T* and becomes zero at *T* > *T*_NI_, again in agreement with the width of the soft plateau being proportional to the nematic order parameter. This confirms that the nematic state with the order parameter *Q*(*T*) ≠ 0 is the cause of the system arrested in strain recovery.

### Microscopic hysteresis on evolution of director distribution

**Fig. 3:**
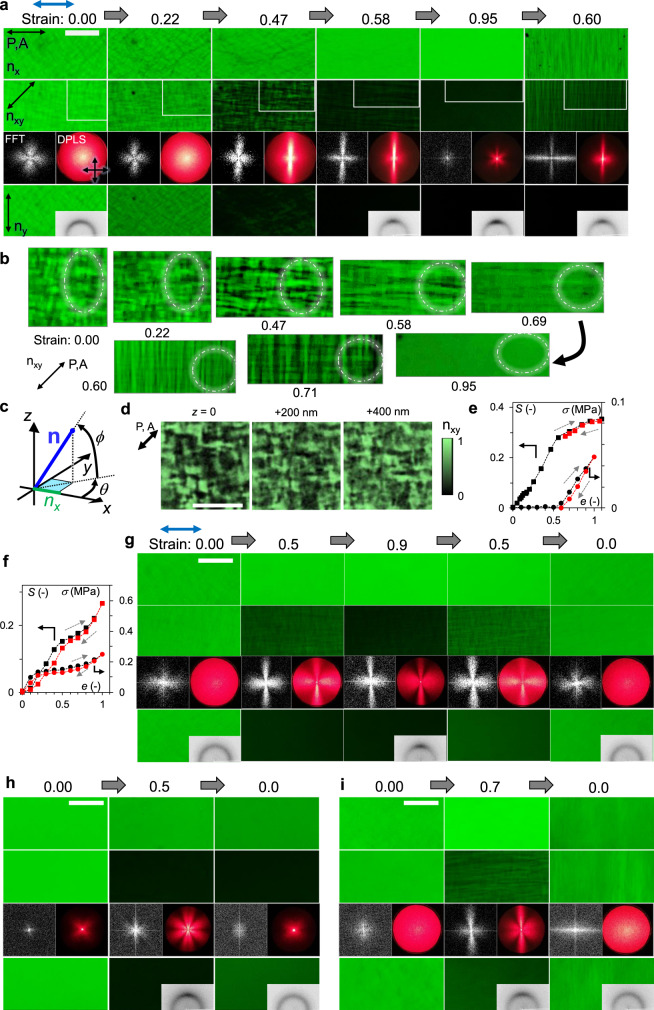
Typical transformation of nematic director patterns. **a**, **b**, **d**, **e** on X1D, **c** definition of the nematic director **n**, **f**, **g** on X1n10, **h** on X3, and **i** on X1i10. On **a**, **g**, **h** and **i**, PFOM images with three different polariser/analyser angles (indicated double headed arrows with *n*_x_, *n*_xy_ and *n*_y_) on a stretching–releasing cycle. Strain is in the *x* direction (blue arrow). (Bar: 10 µm). Each fast Fourier transformed (FFT) on *n*_xy_ image, DPLS pattern with the crossed polarisers (black arrows), and some WAXS patterns are also shown. On **b**, magnified *n*_xy_ images at the location indicated by white rectangular parts on **a**, which is initially 10^2^ μm^2^ in size, with the contrast enhanced. The dashed circles are guides for eyes to trace the domain transformation. **e**, **f** Evolutions of order parameter *S* with corresponding stress–strain curves. The black and red data are of stretching and releasing, respectively. **d** Confocal-PFOM images of initial polydomain taken at the fixed *xy* location and at the different focal plane, *z*, with polarisers in the diagonal direction shown by the arrow. (Bar: 3 µm).

To understand the correlation between the macroscopic hysteresis and the microscopic nematic director domain states, fluorescence microscopy (PFOM) and depolarised light scattering (DPLS) were used to characterise the real and reciprocal space information at the surface and bulk at the micron scale, respectively (Fig. 3). Additionally, the wide-angle-X-ray-scattering (WAXS) gives reciprocal space information of bulk at the molecular scale (Supplementary Fig. 9), which firstly confirm that the all present LCE are in the nematic phase independently of the strain state at 20 °C.

Most of the present LCE at the nematic phase initially show a polydomain texture (Fig. 3a, b at *e* = 0) known as the result of the competition between nematic order that promotes the uniform director alignment, and the quenched random disorder inherently introduced by their network crosslinks^20,27^. While the distribution of the nematic director **n** (Fig. 3c) is macroscopically isotropic (Supplementary Fig. 10), the remarkable short-ranged orientational correlation in ±45° from the polarisers direction appears, which also manifests in their fast Fourier transformed (FFT) images and DPLS patterns. This orientational correlation in polydomain LCE has been theoretically predicted by Uchida^40^. Moreover, the three-dimensional (3D) observation (Fig. 3d), which shows the gradual change in the pattern on changing the focal plane holding the characteristic correlation, clarifies that the polydomain pattern with the characteristic length scale of ~1 µm spans over the bulk and is ubiquitous in the sample.

Upon stretching, the gradual rotation of domains toward the strain axis is directly recognised from the intensity difference between *n*_x_ and *n*_y_ images, in which polarisers are in *x* and *y* directions, respectively (Fig. 3, Supplementary Fig. 11). It is also confirmed by the evolution of macroscopic orientational order parameter *S* with *e* (Fig. 3e for X1D, 3f for X1n10)^26^. Here, *n*_xy_ images with polarisers in the direction +45° from the strain axis are convenient to evaluate how the counter-rotating domains evolve, because they differentiate the sign of director angle *θ* from the strain axis (Fig. 3c). The contrast of *n*_xy_ images initially increases and starts decreasing over the soft range, indicating that both counter-rotating domains are aligning toward the strain axis.

At strain of 0.4~0.6 on X1D (Fig. 3a, b) showing the maximum *n*_xy_ contrast, the higher distribution of *θ* around ±45° is suggested. The corresponding images (Supplementary Fig. 12) show a characteristic lattice-like pattern with two roughly orthogonal principal axes perpendicular to each other, one of which corresponds to the strain axis as also resolved in the 3D image (Supplementary Fig. 13). The corresponding FFT and DPLS patterns with the anisotropic four-leaves pattern are similar to the previously observed one by Clarke et al.^20,39^. Here we reveal it in the real space as the present lattice-like pattern that also expands in 3D. Although PFOM images of some LCE are poorly resolved, FFT of their *n*_xy_ images with DPLS patterns suggest that all LCE show the similar director transformation via the lattice-like structure (Supplementary Fig. 11). The results suggest a scenario (Supplementary Fig. 14), where the domain walls separating the counter-rotating domains are chosen from domain boundaries initially existing everywhere in the polydomain, and grow accompanying their sharpening^27^. The scenario is also compatible with the theoretically proposed textured deformation^23,24^ for soft responses, in which the imposed macroscopic strain change is built entirely out of simple uniaxial stretches with rotations at respective parts.

The point of present interest is whether the domain transformation is reversible or hysteretic through a cycle of stretching–releasing and the correlation to the macroscopic hysteresis. This is most simply evaluated by comparing the PFOM/DPLS images at the same strain upon stretching and releasing. For example, X1n02 and X1n10 show almost reversible transformation (Supplementary Fig. 10, Fig. 3e, f). In contrast, on X1, X1R, X2, X3, X1i02 and X1i10, the lattice-like pattern reappears with noticeable changes after releasing (Supplementary Fig. 10, Fig. 3e). Especially in X1D, the clear stripe pattern forms (Fig. 3a, b, Supplementary Fig. 15), similar one of which is well known in the ‘stripe domain’ transition^19,40–42^ in pre-aligned LCE. This stripe pattern clearly differs from the lattice-like pattern formed upon stretching, suggesting the highest hysteresis among others. Moreover, the stripe pattern is recognisable by naked eyes as it glitters due to the periodic microstructure (Supplementary Movie 1), showing potential as optical elements.

In any present hysteretic cases, depending on the history of how macroscopic strain has been applied, the system state is transferred to different points in the multidimensional director (or polymer chain) configurational space, **Ω** ≡ {***n***_***i***_}, where *i* is the sequential number for unit directors along polymer strands. Note that different **Ω** may be realised under a certain strain *e*, i.e. **Ω**(*e*) is the multivalued function, whereas a certain configuration **Ω** uniquely determines *e*, i.e. *e*(**Ω**) is the single-valued one. In contrast, in reversible systems, X1n02 and X1n10, **Ω**(*e*) appear as a single-valued function.

Interestingly, X3 and X1i10, which show full strain recovery, do not recover the initial director distribution (Fig. 3h, i); final PFOM images of X3 show different fluorescence intensities and the final *n*_xy_ images (and FFT) of X1i10 suggest the lattice-like structure. This indicates that the macroscopic hysteretic behaviour is not always consistent with the microscopic evolution. In such a case, the system can exist at different points in **Ω** even at zero strain, i.e., it is, in principle, possible to manipulate and memorise the director orientation with appropriate mechanical stimuli without invoking macroscopic deformation.

After thermal annealing of LCE with the modulated and arrested director distribution at isotropic temperature (80 °C for ~5 min), the original polydomain structures, **Ω**|_*PD*_, recover (Supplementary Fig. 16). Thus, the observed hysteretic behaviour is not plastic deformation, i.e., the network structure is not broken, but is caused by the anomalous slowing-down of the collective director orientation relaxation **Ω**(*t*) associated with that of the polymer configuration toward the optimal state **Ω**|_*PD*_, the origin of which is discussed next.

### Potential energy landscape view and unfavourable hairpin motion

**Fig. 4:**
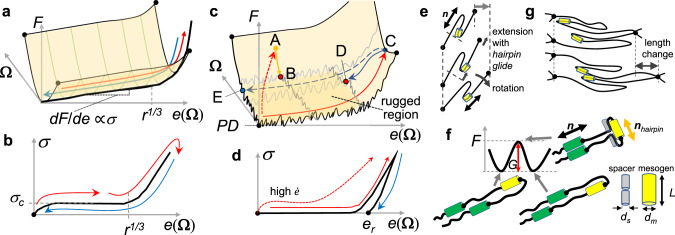
Phenomenological PEL view of reversible and hysteretic LCE systems. **a**, **b** Reversible case showing a typical semi-soft elasticity. **a** PEL, *F* vs. strain *e*(**Ω**). Note that **Ω** is the highly multidimensional vector. **b** Stress–strain curve. The threshold stress ***σ***_***c***_ directly proportional to the slope ***df*/*de*** of the quasi-linear part of ***F***. The soft-elastic range spans ***e*** = ***r***^1/3^, where *r* measures the anisotropy of the average chain shape spheroid. The system is reversible through a cycle of increasing and decreasing ***e*** (arrows). **c**, **d** Hysteretic case. **c** PEL, *F* with a highly rugged region, which can arrest the system. Upon increasing strain (red arrows), the route on PEL may depend on strain history. When the system is equilibrated from point A (or C), the system relaxes to one of the narrowly avoided local minima, point B (or D), where ***dF***/***de*** = ***σ***_***c***_ ~ 0. When ***e***_***r***_ ~ 0, the system may reach E from C. **e** Schematic of hairpin glide associated with soft deformation composed of simple extension (or pure shear) and rotation process. **f** The primitive hairpin glide process that may induce the rugged potential surface. At molecular scales, the hairpin structure in a main-chain network strand with mesogenic molecular units must glide along the director direction. The activation energy cost ***G*** is inevitable because the rigid rod-like mesogenic part, with the local director ***n***_***hairpin***_, must transiently be normal to the surrounding nematic mean field. **g** Schematic of hairpin glides upon the length change at a common crosslink.

Dynamics of the observed reversible and hysteretic cases may be described based on the phenomenological view of PEL^34^, which is the multidimensional surface generated by the system potential energy as a function of local coordinates **Ω**. The present PEL is expressed as *F*[*e*(**Ω**)] that is related to observable stress *σ*(*e*) = d*F*/d*e* (Fig. 4a–d). In the reversible case, single potential minimum at certain strain is expected, where the system point on **Ω** can relax to the energy minimum within the experimentally accessible time of tens of minutes after strain perturbation. Although the relaxation is slower in the nematic phase even in X1n02 and X1n10, there are no barrier that prevents the system from accessing the minimum. Thus, the main mechanical features of this case, including the soft elastic response, may be adequately explained as an elastic problem within the equilibrium regime^24^ with minor consideration for the relaxation dynamics.

In contrast, the hysteretic PEL should have a rugged surface, within which the system can be easily arrested in narrowly defined local minima. The arrested point depends on the trajectory of the system in **Ω** space. Upon stress relaxation after an applied strain jump, as in Supplementary Fig. 3, the system is arrested when it reaches the rugged region (the route from point A to B in Fig. 4c). Upon releasing the load from a highly stretched state, as in the recovery experiments shown in Fig. 2g, h, the system first slides down a slope due to the ordinary elastic return, and then is trapped as soon as it enters into the soft plateau range (from point C to D in Fig. 4c).

The next question is about the mechanism that produces the rugged PEL in our main-chain N-LCE. In the ordinary description of soft elasticity^1,22–25^, the dynamics of the polymer strands has not been treated directly. Here we consider this to seek the origin of the local energy barriers. Since different regions in LCE are required to stretch and rotate differently to locally absorb the imposed strain, as prescribed by the textured deformation model^23,24^, the polymer strands between crosslinks also should do so, which is the primitive process of soft rotation at the molecular scale, as shown in Fig. 4e. As a simple consequence, we notice that the strands must move inherent hairpins to accomplish the required process (in the case of main-chain nematic polymers, side-chain LCE do not have hairpins). Considering the nematic phase promoting mutual alignment of strands and the geometrical ability to form hairpins (*L*_CON_/*L*_*X*D_ shown in Table 1), at least a few hairpins with a size close to the spacer between mesogenic rods may form between crosslinks. Since the rod-like mesogenic units (yellow cylinders in Fig. 4e–g) are rigid, the hairpin folds should occur in the flexible spacer sections, and their glide between the mesogenic parts must encounter an energy barrier (Fig. 4f).

The energy penalty for this single transition state, *G*, may be expressed as *G* = *G*_*nem*_ + *G*_*el*_, involving the nematic and the elastic contributions. Their crude estimations are possible via evaluations of how much their mean fields are violated by the transient state (Supplementary Note 1). As a result, *G*_*nem*_ ~ 26*k*_B_*T* and *G*_*el*_ ~ 0.025*k*_B_*T*, and thus, *G*/*k*_B_*T* ~ *G*_*nem*_/*k*_B_*T* ~ 26 » 1, where *k*_B_ is the Boltzmann constant and *T* = 20 °C. This suggests that the nematic order makes the single hairpin motion a very infrequent event. Consequently, any change in macroscopic strain (Fig. 4g) may require the activation energy input to at least overcome *G*, even though their energy states separated by the barrier are almost the same as deduced from the soft elastic theory in the equilibrium regime^1,22–25^.

Upon increasing strain, the externally acting stress helps this transition, overcoming the local energy barriers. However, during strain recovery driven by the weak internal stress alone, especially within the soft elastic range, director rotations are prohibited by the inactive hairpins, leading to the arrested states. Note that *G* should rapidly decrease with increasing temperature toward *T*_NI_, and becomes zero at *T*_NI_ since the mean field originates from the nematic order *Q*(*T*) (Supplementary Note 1), supporting the full recovery observed at isotropic state (Fig. 2h). Overall, the critical slowing-down of the hairpin glide events restricted by the nematic order is the feasible origin of the rugged PEL in main-chain N-LCE. This effect should be absent in the side-chain N-LCE having no hairpins. This leads to the reversible stress–strain response^30^ explainable in the equilibrium regime^24^. Phenomenologically similar situation is assumed for the present partially nematic genesis LCE, X1n02 and X1n10. In this case, energy input for the hairpin glide is not dissipated and is elastically stored, which manifests as non-zero *σ*_*c*_ and the smaller *m*, and drives the recovery of both director configurations and macroscopic strain upon releasing applied strain.

According to the present model, other pure isotropic genesis main-chain N-LCE generally show some hysteresis with the slower relaxation (higher *m* value), supporting present results (Table 1). On LCE showing the soft elastic range, the macroscopic residual strain with microscopic director patterns different from the initial polydomain appear, corresponding to X1D, X1, X1R, X2, and X1i02. Even those without the soft elastic range due to the higher *n*_XD_, X3 and X1i10, strain history remains as the anisotropic director patterns (point E in Fig. 4d), where the applied strain overwrites the configuration of hairpins in relatively free strands that do not support the macroscopic elasticity. In X1R, *G* should be further increased by more rigid spacer segments, resulting in the even slower relaxation before being arrested. In X1D, for which the highest hairpin density is expected, the higher number of local minima in the rugged region on PEL is expected. Thus, increased flexibility in choosing director distributions freely from the inherent random disorder constraint may induce the regular stripe pattern, which is favourable in terms of the Frank elasticity minimising domain boundaries.

## Discussion

The present findings, via analysis of a series of specially designed LCE, of the internal constraints due to the coupling between the nematic alignment and the polymer network topology through the hairpin dynamics unifies the equilibrium and non-equilibrium features known for decades in N-LCE, namely soft-elasticity^1,19–25^, slow relaxation^28–30^, and hysteresis^43,44^. The result highlights that the balance between elastic and hysteretic properties can be controlled by engineering polymer network design. This is useful in the development of actuation, shape-morphing, damping, and tribological materials. Further, the suggested re-writability of local director orientations via proper thermal/mechanical stimuli also offers reconfigurable optical elements and possibility of crosslinked-polymer-based memory devices.

## Methods

### Materials and preparation of LCE

For preparation of LCE, we slightly modified the methods reported previously^14,45–47^, and single-step crosslinking reaction of a thiol-acrylate Michael addition is used. The diacrylate monomer, 1,4-bis-[4-(6-acryloyloxyhexyloxy)benzoyloxy]-2-methylbenzene (RM82), was purchased from Wilshire Technologies. The diacrylate spacer, tri(propylene glycol) diacrylate (TPGDA, isomer mixture), and three thiol monomers: 2,2′-(ethylenedioxy) diethanethiol (EDDET), 1,4-benzenedimethanethiol (BDMT) and pentaerythritol tetrakis (3-mercaptopropionate) (PETMP), were purchased from Sigma Aldrich. Triethylamine (TEA, Sigma Aldrich) was used as the catalyst of the Michael-addition thiol-ene reaction. As the radical scavenger, butylated hydroxytoluene (BHT, from Sigma Aldrich) was used to supress the unwanted radical polymerisation reaction between acrylates. Irgacure2959 (Sigma Aldrich) was used as the photoinitiator for LCE with excess acrylates. As the dichroic fluorescent probe, coumarin 545 T (C5T, Tokyo Chemical Industry)^48,49^ was used. Dimethylformamide (DMF, Sigma Aldrich) and toluene (Sigma Aldrich) were used as the solvents. All chemicals were used in their as-received condition with no purification.

At the specific molar ratio of functional groups shown in Supplementary Table S1, RM82, TPGDA, EDDET (or BDMT) and PETMP were weighed, BHT (0.5 wt%) and Irgacure2959 (only for LCE with excess acrylates at 0.2 wt%) were added, DMF was added to at 5 wt%, and toluene was added at 50 wt% only for X1D. Note that the more amount of solvent, the less entanglement was expected^50^. After each mixture was gently mixed at an elevated *T* ~ 70 °C for ~10 min, TEA was added at 1.5~3 wt% to start the Michael-addition reaction between thiol and acrylate groups. The mixture was kept between two glass slides with the spacer with 0.7~0.9 mm at 75 °C (isotoropic phase) overnight. The sample was released from the glass mold, and placed in the vacuum oven at 75 °C overnight for de-swelling. For the samples with toluene, which showed larger volumetric shrinkage, the de-swelling was done by floating it on the hot water surface to minimise the external constraint before drying in dried vacuum oven. LCE with excess acrylates were then photopolymerised by irradiating ultraviolet (UV) light (365 nm, 200 mm from the lamp source, 8 W, LSUV-8, Azone) for 20 min. For X1i02 and X1i10, the UV was irradiated at 75 °C (isotropic). For X1n02 and X1n10, it was done at 20 °C (nematic), which resulted in partially nematic genesis LCE.

The nominal number density of the crosslinks per unit volume, *n*_XD_, was calculated using each molar ratio with the molecular weights of monomers (Supplementary Table S1). The nominal distance between cross-links, $L_{{{{\mathrm{XD}}}}} = \root {3} \of {{1/n_{XD}}}$. *L*_CON_ is the contour length of a nominal strand between nearest neighbouring crosslinks. Assuming that the approximate lengths of each monomers, as 4.3, 2.1, 1.4, 1.0 and 0.8 nm for RM82, TPGDA, PETMP, EDDET and BDMT, *L*_CON_ were estimated from the nominal ratio of monomer components (Supplementary Table S1). *L*_CON_/*L*_XD_ is an index of the ability to form hairpins. The lower effective crosslink density is expected for X1D than that of X1 due to the difference in entanglements that effectively work as crosslinks^51^. The present estimated values for excess acrylate systems are crudely calculated by setting that three excess acrylates make a crosslink point. Although this may be an overestimation, their trends on comparison to X1 would be correct.

To dye the sample, a 5 µL droplet of the 0.2 wt% DMF solution of C5T was cast on he surface at 75 °C, and then, was placed in a vacuum oven at 75 °C ~6 h to ensure the solvent evaporation prior to the characterisation. Although, alternativelly, it was possible to dye the whole sample by mixing the solution before the polymerisation, the image contrast of the microscopy was not enough for observation of nematic domains. Since only the layer close to the surface was assumed to be dyed with the present post-dying method, in which the background fluorescence intensity could be greatly reduced, this was adopted for the domain characterisation. Although the homogeneity of the probe concentration would not be controlled by the post-dying method, it did not disturb the observation at the present area of interest (~30^2^ µm^2^) arround the centre of the cast location. The dye concentration was estimated via the comparison of the fluorescence intensities of the post-dying to pre-dying sample with the same setup, to be less than 0.02 wt%.The typical sample strip size for stretching experiments was 40 × 5 × (0.9~0.7) mm^3^. No noticable effect of dying on the mechanical properties were found at the present low dye concentration.

### Stress–strain responses

The stress–strain curves for LCE films on the tensile mode were obtained using a commercial instrument (EMX1000, IMADA). In this study, we used the nominal strain *e* = (*L* − *L*_0_)/*L*_0_, where *L* and *L*_0_ are effective and the initial length of the sample strip, respectively, and nominal stress *σ*, which is the force reading divided by the initial cross sectional area. The LCE strips width, thickness and effective length were 5 mm, 0.6~0.8 mm and 30 mm, respectively. Equilibrated stress–strain curves were obtained by applying the strain jump of 0.1 followed by equilibration time of 300~1000 s. Stress–strain curves at constant strain rates were also taken. For sample break measurements, the strain was increased until the sample break at the rate of extension of 0.00042 s^−1^.

### Strain recovery tests

LCE strips hung by clumping at one end were first strained up to a certain strain and held for 100 s. Then, the strain was abruptly released to allow the spontaneous shrinkage, which was monitored. The experiments were conducted in a temperature-controlled space. For estimation of nematic–isotropic transition temperature LCE strips were hung with a certain weight that applied load and the length were monitored upon elevating temperature at ~1 °C/min. Assuming that the present nematic LCE show weak first order transition, which may become supercritical under stress, temperature that gives the maximum slope was adopted as nematic–isotropic transition temperature.

### Polarised fluorescence optical microscopy

To observe fluorescent images, we used a conventional reflection-mode fluorescence optical microscope (FOM), where the light source was mounted above the sample and the excitation light passed through the microscope objective lens on its way toward the sample. A Xe lamp (75 W) was used as the light source. To detect the emission from fluorescence probe, we used a fluorescence filter set (U-MWIB-3, Olympus) comprising an excitation filter that transmitted light with wavelengths between 460 nm and 495 nm, and an emission filter that transmitted light with wavelength larger than 510 nm. The images were collected at the pixel size of 0.154 µm using a Nikon DS-Ri2 camera connected to a computer and controlled through imaging software (NIS-Elements, Nikon). An objective lens with a numerical aperture (NA) of 0.75 (MPLFLN-BDP50x, Olympus) was mainly used. The strain was applied with the homemade stretcher. The images were taken at various and the average strain rate was ~0.0004 s^−1^.

To observe the nematic domains the linearly polarised excitation was used and the polarised fluorescence intensity in the identical polarisation was measured (PFOM). The focus was carefully adjusted to the top surface, where the contrast became highest. With the positive dichroic dye, which is used here, the dichroic contrast is enhanced with this setup^49^. On PFOM images, the brighter parts indicate that the absorption and fluorescence transition dipole moment (TDM) vectors of the probe have the more components in the polariser direction. Since the present dye molecules align parallelly to the nematic directors of the LCE, which was confirmed by the positive dichroic ratio under uniaxial stretching, the domains observed on the PFOM images directly reflect the polydomain structure in two-dimension. Given the polarisers along the *x* axis, the fluorescence intensity reflects the *x* component *n*_*x*_ of the director **n**. If the polarisers (or the sample) is rotated, the brightest or darkest part gradually changes the intensity and becomes darker or brighter, respectively.

### Macroscopic order parameter estimation

The macroscopic order parameter of the nematic directors of LCE with respect to the strain axis, *S*, was estimated using dichroic ratio, *R*, of the fluorescence intensity over the observed area. *I*_‖_ and *I*_⊥_ are the intensities with the polarisers parallel and perpendicular to the strain axis, respectively. Using the obtained *R* = (*I*_max_/*I*_min_)^1/2^, where the power of ½ is required for the case using polarisers for both excitation and emission^49^. Then, *S*_TDM_, which is the order parameter of the fluorescence TDM of the probe aligning along nematic directors, can be calculated as $S_{{{{\mathrm{TDM}}}}} = \frac{{R - 1}}{{R + 2}}$. Note that the absolute value of *S*_TDM_ is problematic to be used as *S* due to the background noise and non-parallel light geometry due to the lens, and thus, should be less than *S* because the free fluorescent probes should fluctuate more. The reliable value of order parameter *S* may be obtained using WAXS, and thus, *S*_TDM_ were corrected using the WAXS-originated *S* obtained at higher strain, as described at the WAXS method section below.

### Three-dimensional (3D) PFOM by confocal laser scanning microscopy

A confocal laser scanning fluorescence microscope (A1^+^ system, Nikon) was used to obtain confocal PFOM images of polydomain sample at 25 °C (nematic) with the higher 3D optical resolutions. An optically pumped semiconductor laser (LU-N4 Laser Unit, Nikon, equipped with Sapphire 488, Coherent Inc.) was used to excite the fluorescent probes at 488 nm and the emitted light between 525 and 595 nm was collected. An objective lens with an NA of 0.90 (TU PlanFluor100 ×, Nikon) was used. The confocal PFOM images were acquired at the voxel size of typically 30(*x*) × 30(*y*) × 50(*z*) nm^3^. The excitation laser was linearly polarised and emitted fluorescence in the same polarisation was collected using a polariser. The obtained images were properly deconvoluted using the associated software. In the present LCEs with dyes, image slices from the top surface to a few µm depth were properly taken with sufficient fluorescent intensity.

### Depolarised light scattering (DPLS)

For DPLS measurement, a He-Ne laser (wavelength of 632.8 nm, 1 mW, Melles Griot 05-LHP-111) was used. The sample was placed between a set of polarisers under crossed-nicol condition, which is often called as the HV mode. The diameter of the laser light was ~0.6 mm. The scattering pattern on a paper screen placed at the distance of 20–40 mm from the sample was captured by a digital camera. The same position on the sample, at which the optical microscopy was performed. To minimise the effect of the birefringent due to the alignment, the strain axis was set to parallel to the polariser direction. With this configuration, the scattering from the birefringence components that are not along the orthogonal polariser directions^20^.

### Dynamic scanning calorimetry (DSC)

For differential scanning calorimetry (DSC, DSC4000 PerkinElmer or DSC6100 SII Nanotechnology), samples with approximately 15 mg were loaded into standard aluminium DSC pans. The samples were heated to 100 °C at 10 °C/min, held isothermally for 5 min, and cooled to −50 °C followed by heating up to 100 °C at 5 °C/min to acquire the data. Although the nematic–isotropic transition peak can be ideally found as the endothermic peak, some LCE only show the inflection points, and thus, those were used as the estimates in addition to those obtained by shape recovery experiments (Supplementary Fig. S2). The upper limit of glass transition temperature, *T*_g_^*^, were estimated because the whole shoulder was not obtained. The LCE (X1 and X1D) at the arrested state, which was obtained after application of high strain, were also tested after ~10^6^ s. On this case, the samples were first cooled to −50 °C and then to 100 °C at 5 °C/min to acquire the data, to confirm that there was no new peak for relaxation of crystal or smectic phases.

### Wide angle X-ray scattering (WAXS)

The alignment of LCE at RT was characterised using a Rigaku type 4037 diffractometer using graded d-space elliptical side-by-side multilayer optics, monochromated Cu Kα radiation (wavelength 0.1542 nm, 40 kV, 30 mA), and a flat camera with an imaging plate (Rigaku R-Axis IV) at room temperature. The exposure time was 5 min with a 150 mm sample-to-camera length. The order parameter *S* was estimated from the azimuthal angle, *ψ*, dependent scattering intensity *I*(*ψ*), via $S = {\int}_0^{\pi /2} {I\left( \psi \right)} P_2(\cos \psi )\sin \psi {{{\mathrm{d}}}}\psi /( - \frac{1}{2}{\int}_0^{\pi /2} {I\left( \psi \right)} \sin \psi {{{\mathrm{d}}}}\psi )$, where *P*_2_ is the second order the Legendre polynomial and *ψ* is the angle with respect to the applied uniaxial strain direction. Assuming the WAXS-derived *S* is more reliable, the values at the high strain of 0.6~1.0, at which the nematic directors are clearly aligned, were used to correct those estimated by *S*_TDM_ obtained from the fluorescent dichroism.

## Supplementary information

Supplementary Information

Description of additional supplementary information file

Supplementary Movie 1

## Data Availability

Data that support the findings of this study are available from the corresponding author upon reasonable request.

## References

[1] Warner, M. & Terentjev, E. M. *Liquid Crystal Elastomers* (Oxford Univ. Press, 2007).

[2] White TJ, Broer DJ (2015). Programmable and adaptive mechanics with liquid crystal polymer networks and elastomers. Nat. Mater..

[3] Camacho-Lopez M, Finkelmann H, Palffy-Muhoray P, Shelley M (2004). Fast liquid-crystal elastomer swims into the dark. Nat. Mater..

[4] Pei Z (2013). Mouldable liquid-crystalline elastomer actuators with exchangeable covalent bonds. Nat. Mater..

[5] Ohm C, Brehmer M, Zentel R (2010). Liquid crystalline elastomers as actuators and sensors. Adv. Mater..

[6] Ware TH, Biggins JS, Shick AF, Warner M, White TJ (2016). Localized soft elasticity in liquid crystal elastomers. Nat. Commun..

[7] Guin T (2018). Layered liquid crystal elastomer actuators. Nat. Commun..

[8] Rousseau IA, Mather PT (2003). Shape memory effect exhibited by smectic-C liquid crystalline elastomers. J. Am. Chem. Soc..

[9] Ishige R (2008). Elongation behavior of a main-chain smectic liquid crystalline elastomer. Macromolecules.

[10] Patil HP, Lentz DM, Hedden RC (2009). Necking instability during polydomain-monodomain transition in a smectic main-chain elastomer. Macromolecules.

[11] Ren W, Griffin AC (2012). Mechanism of strain retention and shape memory in main chain liquid crystalline networks. Phys. Status Solidi Basic Res..

[12] Clarke SM (2001). Soft elasticity and mechanical damping in liquid crystalline elastomers. J. Appl. Phys..

[13] Azoug A (2016). Viscoelasticity of the polydomain-monodomain transition in main-chain liquid crystal elastomers. Polymer.

[14] Ohzono T, Saed MO, Terentjev EM (2019). Enhanced dynamic adhesion in nematic liquid crystal elastomers. Adv. Mater..

[15] Ohzono T, Norikane Y, Saed MO, Terentjev EM (2020). Light-driven dynamic adhesion on photosensitized nematic liquid crystalline elastomers. ACS Appl. Mater. Interfaces.

[16] Ohzono T, Saed MO, Yue Y, Norikane Y, Terentjev EM (2020). Dynamic manipulation of friction in smart textile composites of liquid-crystal elastomers. Adv. Mater. Interfaces.

[17] de Gennes, P. G. & Prost, J. *The Physics of Liquid Crystals* (Oxford Univ. Press, 1993).

[18] de Gennes, P. G. in *Polymer Liquid Crystals* (eds Cifferi, H. von A., Krigbaum, W. R. & Meyer, R. B.) (Academic Press, 1982).

[19] Kundler I, Finkelmann H (1995). Strain-induced director reorientation in nematic liquid single crystal elastomers. Macromol. Rapid Commun..

[20] Clarke SM, Terentjev EM, Kundler I, Finkelmann H (1998). Texture evolution during the polydomain-monodomain transition in nematic elastomers. Macromolecules.

[21] Urayama K, Mashita R, Kobayashi I, Takigawa T (2007). Stretching-induced director rotation in thin films of liquid crystal elastomers with homeotropic alignment. Macromolecules.

[22] Warner M, Bladon P, Terentjev EM (1994). “Soft elasticity” — deformation without resistance in liquid crystal elastomers. J. Phys. II.

[23] DeSimone A, Dolzmann G (2002). Macroscopic response of nematic elastomers via relaxation of a class of SO(3)-invariant energies. Arch. Ration. Mech. Anal..

[24] Biggins JS, Warner M, Bhattacharya K (2009). Supersoft elasticity in polydomain nematic elastomers. Phys. Rev. Lett..

[25] Golubovic L, Lubensky TC (1989). Nonlinear elasticity of amorphous solids. Phys. Rev. Lett..

[26] Schätzle J, Kaufhold W, Finkelmann H (1989). Nematic elastomers: the influence of external mechanical stress on the liquid-crystalline phase behavior. Makromol. Chem..

[27] Fridrikh SV, Terentjev EM (1999). Polydomain-monodomain transition in nematic elastomers. Phys. Rev. E.

[28] Clarke SM, Tajbakhsh AR, Terentjev EM, Warner M (2001). Anomalous viscoelastic response of nematic elastomers. Phys. Rev. Lett..

[29] Hotta A, Terentjev EM (2001). Long-time stress relaxation in polyacrylate nematic liquid crystalline elastomers. J. Phys. Condens. Matter.

[30] Urayama K, Honda S, Takigawa T (2006). Slow dynamics of shape recovery of disordered nematic elastomers. Phys. Rev. E.

[31] Beyer P, Terentjev EM, Zentel R (2007). Monodomain liquid crystal main chain elastomers by photocrosslinking. Macromol. Rapid Commun..

[32] Lubensky TC, Terentjev EM, Warner M (1994). Layer-network coupling in smectic elastomers. J. Phys. II.

[33] Osborne MJ, Terentjev EM (2000). Elasticity of rubber with smectic microstructure. Phys. Rev. E.

[34] Goldstein M (1969). Viscous liquids and the glass transition: a potential energy barrier picture. J. Chem. Phys..

[35] Debenedetti PG, Stillinger FH (2001). Supercooled liquids and the glass transition. Nature.

[36] Cao P, Short MP, Yip S (2019). Potential energy landscape activations governing plastic flows in glass rheology. Proc. Natl Acad. Sci. USA.

[37] Leeson DT, Gai F, Rodriguez HM, Gregoret LM, Dyer RB (2000). Protein folding and unfolding on a complex energy landscape. Proc. Natl Acad. Sci. USA.

[38] Adams JM, Warner M (2005). Hairpin rubber elasticity. Eur. Phys. J. E.

[39] Clarke SM, Nishikawa E, Finkelmann H, Terentjev EM (1997). Light-scattering study of random disorder in liquid crystalline elastomers. Macromol. Chem. Phys..

[40] Uchida N (2000). Soft and nonsoft structural transitions in disordered nematic networks. Phys. Rev. E.

[41] Verwey GC, Warner M, Terentjev EM (1996). Elastic instability and stripe domains in liquid crystalline elastomers. J. Phys. II.

[42] Conti S, DeSimone A, Dolzmann G (2002). Semisoft elasticity and director reorientation in stretched sheets of nematic elastomers. Phys. Rev. E.

[43] Tokita M, Tagawa H, Niwano H, Osada K, Watanabe J (2006). Temperature-induced reversible distortion along director axis observed for monodomain nematic elastomer of cross-linked main-chain polyester. Jpn. J. Appl. Phys..

[44] Saed MO, Torbati AH, Nair DP, Yakacki CM (2016). Synthesis of programmable main-chain liquid-crystalline elastomers using a two-stage thiol-acrylate reaction. J. Vis. Exp..

[45] Nair DP (2012). Two-stage reactive polymer network forming systems. Adv. Funct. Mater..

[46] Yakacki CM (2015). Tailorable and programmable liquid-crystalline elastomers using a two-stage thiol-acrylate reaction. RSC Adv..

[47] Saed MO (2017). High strain actuation liquid crystal elastomers via modulation of mesophase structure. Soft Matter.

[48] Chen CH, Tang CW (2001). Efficient green organic light-emitting diodes with stericly hindered coumarin dopants. Appl. Phys. Lett..

[49] Ohzono T, Yatabe T, Wang C, Fukazawa A, Yamaguchi S (2018). Negative fluorescence anisotropy of phosphole oxide-based dyes in nematic liquid crystals. Commun. Chem..

[50] Urayama K, Kohjiya S (1996). Crossover of the concentration dependence of swelling and elastic properties for polysiloxane networks crosslinked in solution. J. Chem. Phys..

[51] Kutter S, Terentjev EM (2001). Tube model for the elasticity of entangled nematic rubbers. Eur. Phys. J. E.

