# Supplementary material for: Internal constraints and arrested relaxation in main-chain nematic elastomers
Source: Nat Commun. 2021 Feb 4;12:787. doi: 10.1038/s41467-021-21036-3 (PMC7862651; doi:10.1038/s41467-021-21036-3)
Supplement: Supplementary file 1 — Supplementary Information [file 41467_2021_21036_MOESM1_ESM.pdf]

Supplementary Information for

Internal constraints and arrested relaxation in main-chain nematic elastomers

Takuya Ohzono, Kaoru Katoh, Hiroyuki Minamikawa, Mohand O. Saed, Eugene M. Terentjev

- **Supplementary Fig. 1, Main-chain nematic liquid crystal elastomers (LCE).**
- **Supplementary Table 1. Nominal monomer compositions.**
- **Supplementary Fig. 2. Estimation of nematic-isotropic transition temperature T_{NI} shown in Table 1 via thermal and mechanical analysis.**
- **Supplementary Fig. 3. Typical raw data for equilibrated stress-strain curves.**
- **Supplementary Fig. 4. Stress-strain data at a constant strain rate of 0.00083 s^{-1}.**
- **Supplementary Fig. 5. Stress-strain data at a constant strain rate of 0.00083 s^{-1}.**
- **Supplementary Fig. 6. Stress-strain data at a constant strain rate of 0.00042 s^{-1}.**
- **Supplementary Fig. 7. Strain-rate dependence of stress at a certain strain e_S.**
- **Supplementary Fig. 8. Strain recovery curves.**
- **Supplementary Fig. 9. WAXS of LCEs.**
- **Supplementary Fig. 10. PFOM images of polydomain structure of X1 at strain *e* = 0 on sample rotation.**
- **Supplementary Fig. 11. Nematic domain structures through strain cycle.**
- **Supplementary Fig. 12. PFOM images of typical lattice-like domain structure at strain of *e* = 0.6 on sample rotation (X1).**
- **Supplementary Fig. 13. Three-dimensional (3D) lattice-like domain structure at strain of *e* = 0.6 imaged by confocal PFOM (X1).**
- **Supplementary Fig. 14. Two-dimensional expression of the domain transformation scenario.**
- **Supplementary Fig. 15. PFOM images of stripe domain structure at strain of 0.6 after des-stretching on sample rotation (X1D).**
- **Supplementary Fig. 16. PFOM images at 20 °C of initial and final samples after a stretching-releasing cycle followed by annealing at 80°C for 5 min at the same locations.**
- **Supplementary Note 1. Estimation of the energy potential barrier $G = G_{nem} + G_{el}$ for the transition state upon hairpin glide.**
- **References in Supplementary Information**

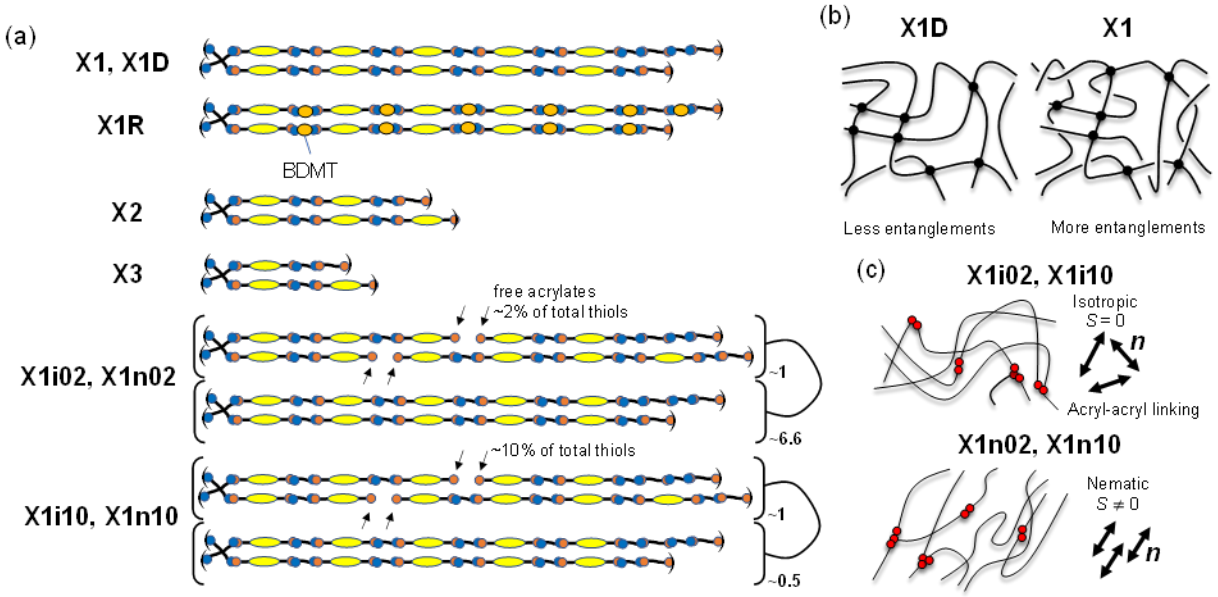

Supplementary Figure 1. Main-chain nematic liquid crystal elastomers (LCE). (a) Schematics of nominal polymer units. Note that the actual strand lengths between crosslinks should have distribution around this picture. Each cartoon of the monomer unit corresponds to that shown in Fig. 1a. For LCEs with excess acrylates, the units before second cross-linking are shown here. (b) Schematics for difference between X1D and X1, showing different density of entanglements. (c) Schematics for the second cross-linking between excess acrylates in the different phases, isotropic (with the order parameter Q = 0) or nematic ($Q \neq$ 0, with a polydomain structure*)*.

X1 is the base system. The cross-linker concentration was doubled or quadrupled in X2 or X3. X1D was cross-linked with solvent at ~55wt% and de-swollen. In X1R, the rigid thiol spacer BDMT was used instead of EDDET. In X1i02, X1i10, X1n02 and X1n10, the total acrylate molar ratio is larger than that of thiol. The excess acrylates are photo-polymerised after the first cross-linking reaction. X1i02 and X1i10 are photo-polymerised at 75°C (isotropic), which was the same temperature used for first reaction. Meanwhile, X1n02 and X1n10 are photo-polymerised at 20°C (nematic) to introduce the memory of polydomain (PD) states. The numbers, 02 or 10, indicate the excess-% amount of the acrylates.

Supplementary Table 1. **Nominal monomer compositions.** The nominal number density of the crosslinks per unit volume, n_{XD}, shown in Table 1, was calculated using each molar ratio with the molecular weights of monomers, which are 672.76, 300.35, 488.66, 182.3, and 170.29 g/mol for RM82, TPGDA, PETMP, EDDET, and BDMT, the density on the order of ~1 g/cm^3 and Avogadro's constant 6.02 × 10^{23} mol^{-1}. The values of fmol-acrylate and fmon-thiol are proportional to the numbers of functional groups, i.e., acrylate and thiol, per nominal network unit (or volume), respectively, which are provided by each monomer. Note that the excess acrylates are finally consumed via photo-polymerisation and linked, which may result in the mixture of simple links and multi-armed cross-links depending on the effective density and mobility of active acrylates. In actual case, the non-reacted dangling bonds may remain. Solvents are evaporated after the first crosslinking reaction (Methods).

		X1D	X1	X1R	X2	X3	X1i02	X1i10	X1n02	X1n10
molar ratio normalised by PETMP	RM82	10.00	10.00	10.00	5.00	2.50	10.20	11.00	10.20	11.00
	TPGDA	3.33	3.33	3.33	1.67	0.83	3.40	3.67	3.40	3.67
	PETMP	1.00	1.00	1.00	1.00	1.00	1.00	1.00	1.00	1.00
	EDDET or BDMT*	11.33	11.33	11.33*	4.67	1.33	11.33	11.33	11.33	11.33
fmol-acrylate	RM82	20.00	20.00	20.00	10.00	5.00	20.40	22.00	20.40	22.00
	TPGDA	6.67	6.67	6.67	3.33	1.67	6.80	7.33	6.80	7.33
fmol-thiol	PETMP	4.00	4.00	4.00	4.00	4.00	4.00	4.00	4.00	4.00
	EDDET or BDMT*	22.67	22.67	22.66*	9.33	2.67	22.67	22.67	22.67	22.67
(excess acrylate)/(thiol) [%]		0	0	0	0	0	2	10	2	10
(solvent)wt% upon 1st crosslinking (solvents)		55 (tolene, DMF)	5 (DMF)	5 (DMF)	5 (DMF)	5 (DMF)	5 (DMF)	5 (DMF)	5 (DMF)	5 (DMF)

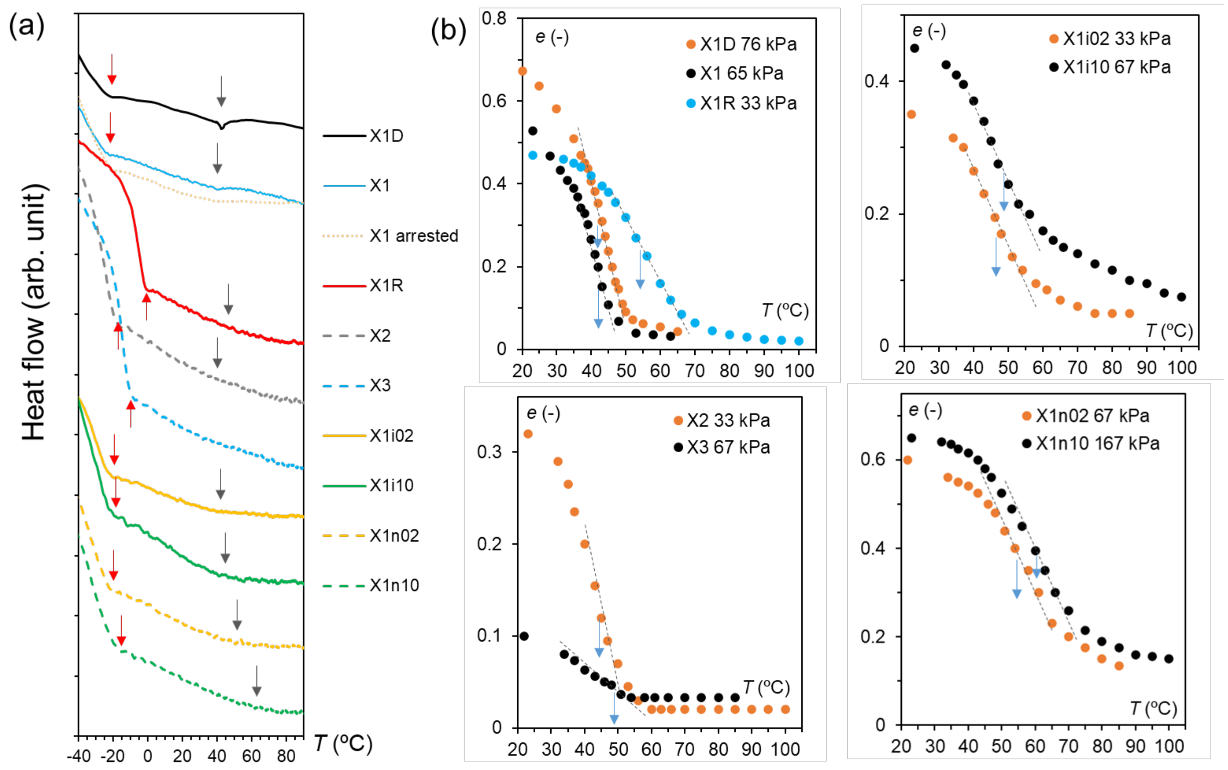

Supplementary Figure 2. Estimation of nematic-isotropic transition temperature T_{NI} shown in Table 1 via thermal and mechanical analysis. (a) DSC curves. In some sample the transition peak appears poorly, only showing the inflection point (indicated by grey arrow) adopted as T_{NI}. For X3, even the inflection point was hardly found. At lower temperature range, a shoulder or the part due to the glass transition appear. "X1 arrested" is the first heating curve of the arrested state with residual strain of 0.45, showing no new peaks attributable to crystal/smectic phases. The whole shoulder corresponding to the glass transition was not obtained. Thus, the upper limit of glass transition temperature, T^*_g, was read at the inflection points at lower temperature range (indicated by red arrow). (b) Temperature-dependent tensile strain *e* under constant stress *s*, which is shown in legends. Assuming that the present nematic LCEs show weak first order transition, which may become supercritical under stress, temperature that gives the maximum slope (indicated by blue arrow) is adopted as T_{NI}. The values are roughly the same as those of DSC, suggesting that the present estimates are correct with possible errors of a few ºC.

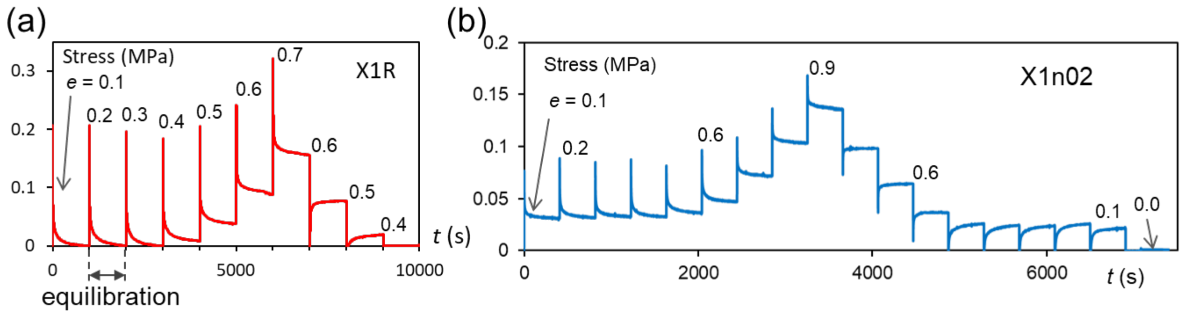

Supplementary Figure 3. Typical raw data for equilibrated stress-strain curves. As examples of the raw data for these stress-relaxation curves of (a) X1R and (b) X1n02, the stress evolution with time are also shown, where the sharp stress increases are results of the stepwise strain increase/decrease and are followed by equilibration.

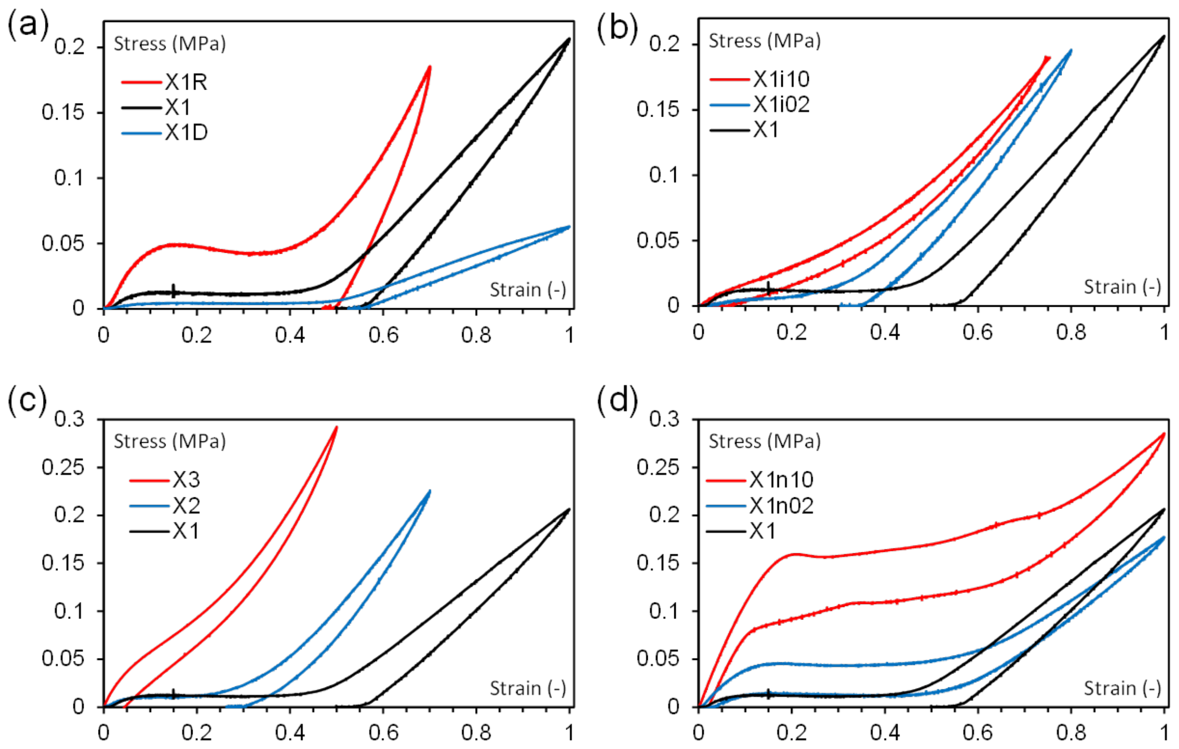

Supplementary Figure 4. Stress-strain data at a constant strain rate of 0.00083 s^{-1}. The apparent non-zero stress at the soft-plateau range are found in X1, X1D, X1R, X2 and X1i02, which relaxed to almost zero after equilibration.

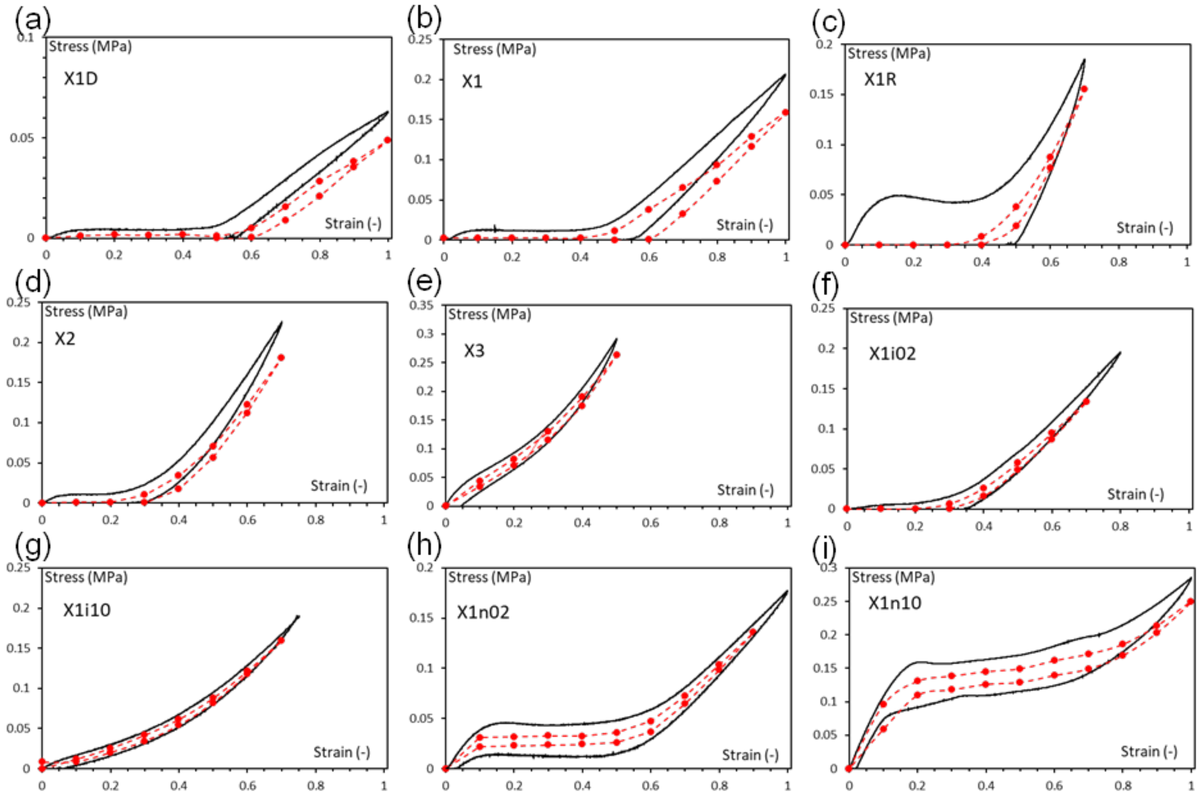

Supplementary Figure 5. Stress-strain data at a constant strain rate of 0.00083 s^{-1}. These are same data shown in Supplementary Fig. 3, for comparison to the equilibrated data (Fig. 2a-d) are also shown (red).

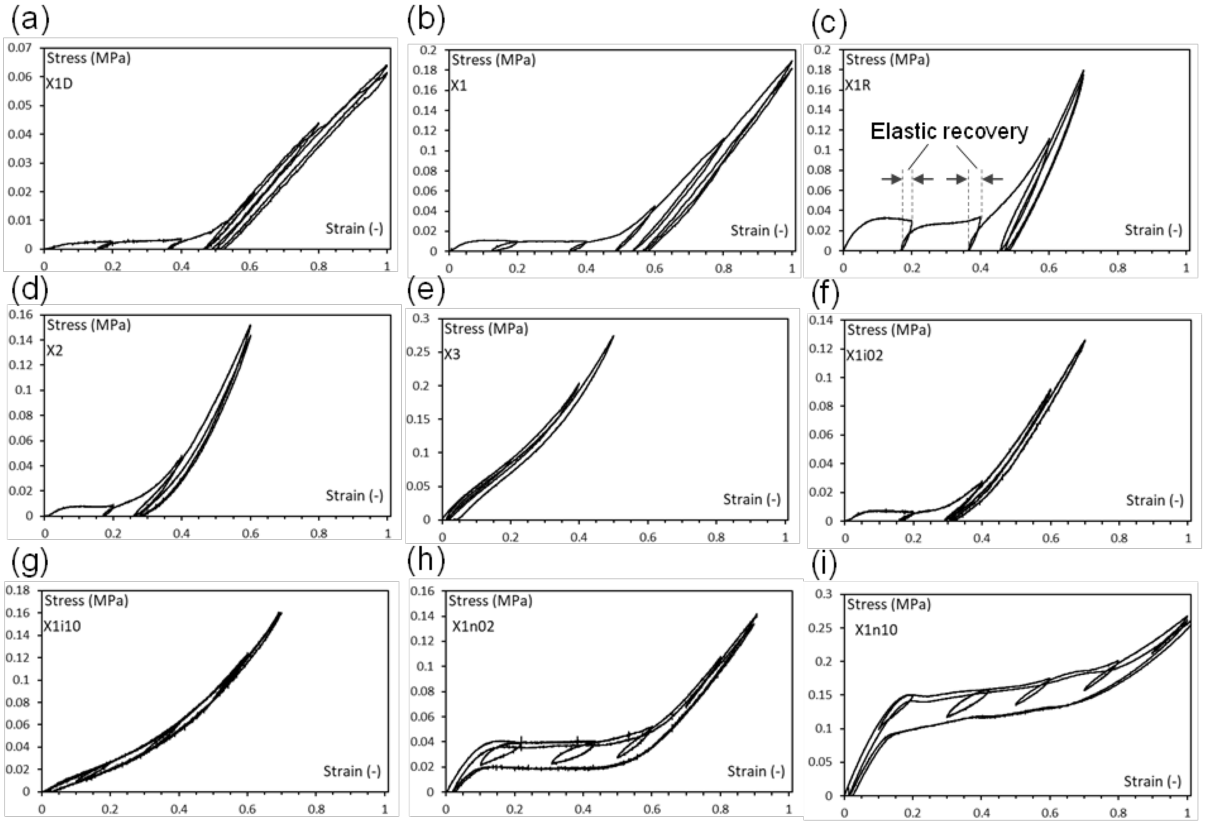

Supplementary Figure 6. Stress-strain data at a constant strain rate of 0.00042 s^{-1}. The strain was repeatedly increased and decreased with different manners. Small elastic components are recognised upon decreasing strain even at the soft range, showing finite slopes. This temporal elastic energy stored at the soft range is finally dissipated via the structural relaxation, as shown in the equilibrated stress-strain curves (Fig. 2a-d). Beyond the soft range, the elastic recovery is found.

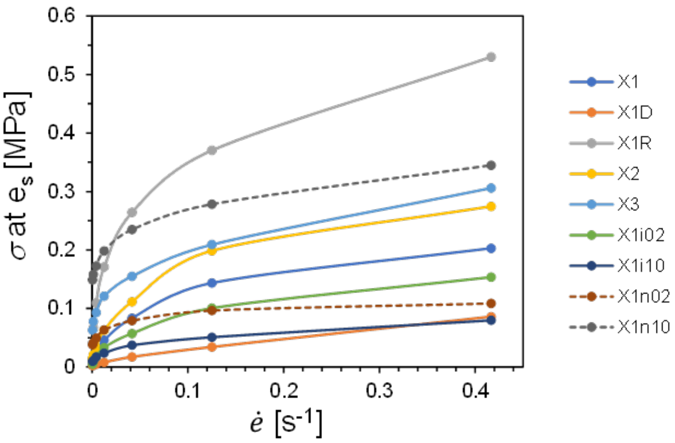

Supplementary Figure 7. Strain-rate dependence of stress at a certain strain e_s. e_s is chosen (Table 1) at soft elastic range at T = 20°C. The same data as in Fig. 2e are shown with normal scales here. Note that all curves show shear-thinning-like response. Especially, X1n02 and X1n10 show the slightly higher degree of the shear thinning, which appear as lower values of *m* as shown in Table 1 and Fig. 2f, suggesting that they behave more elastically than others

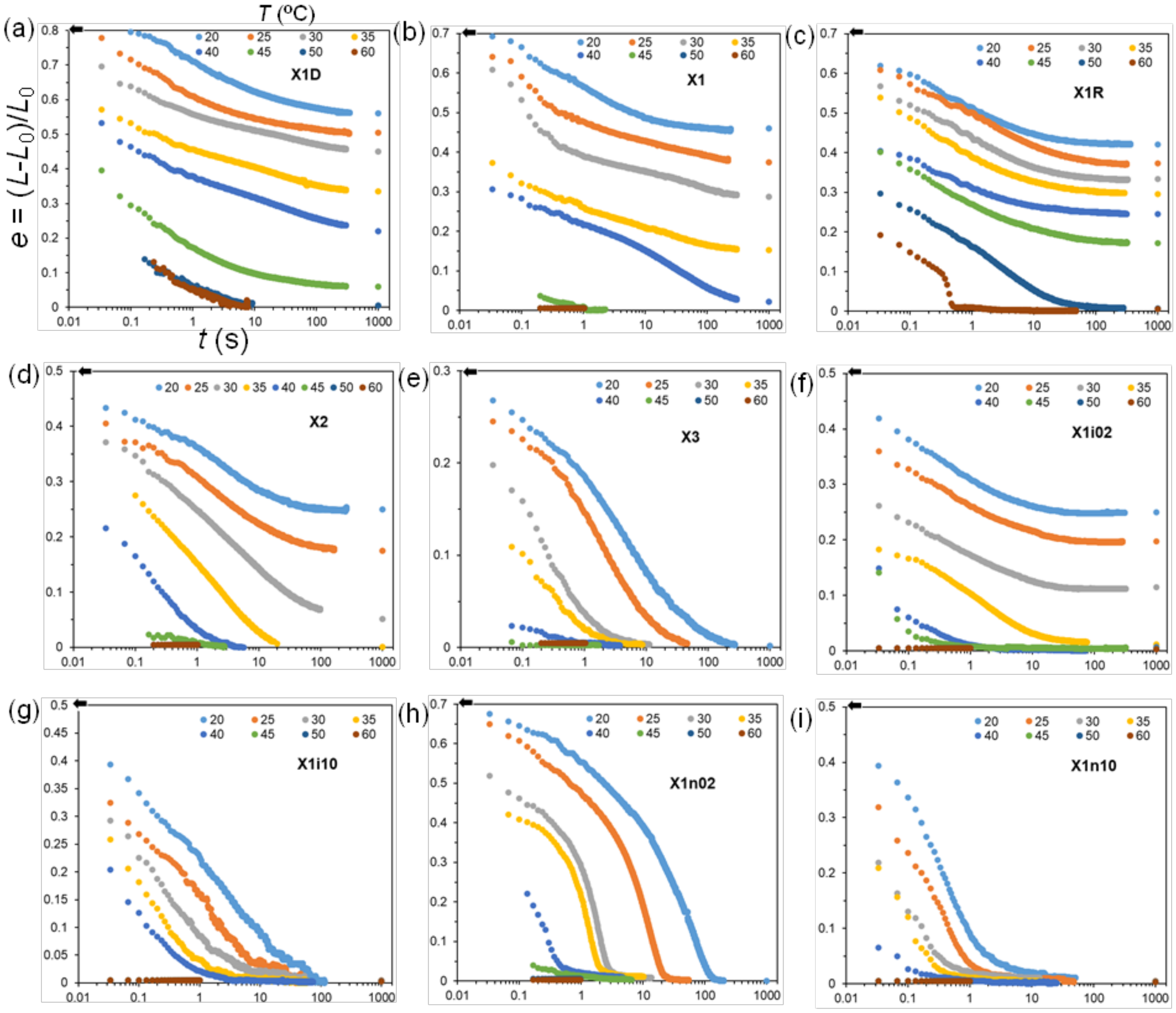

Supplementary Figure 8. Strain recovery curves. The strain relaxation data after a sudden release from an imposed strain, indicated by thick arrows at each upper--left) at various temperature T for present LCE. The samples were hung at one end, and thus, small self-weight was imposed, which is estimated at the clump, ~0.3 kPa. Note that all LCE recovers original shape at T = 60 ºC, at which LCE other than X1i10 are in isotropic phase.

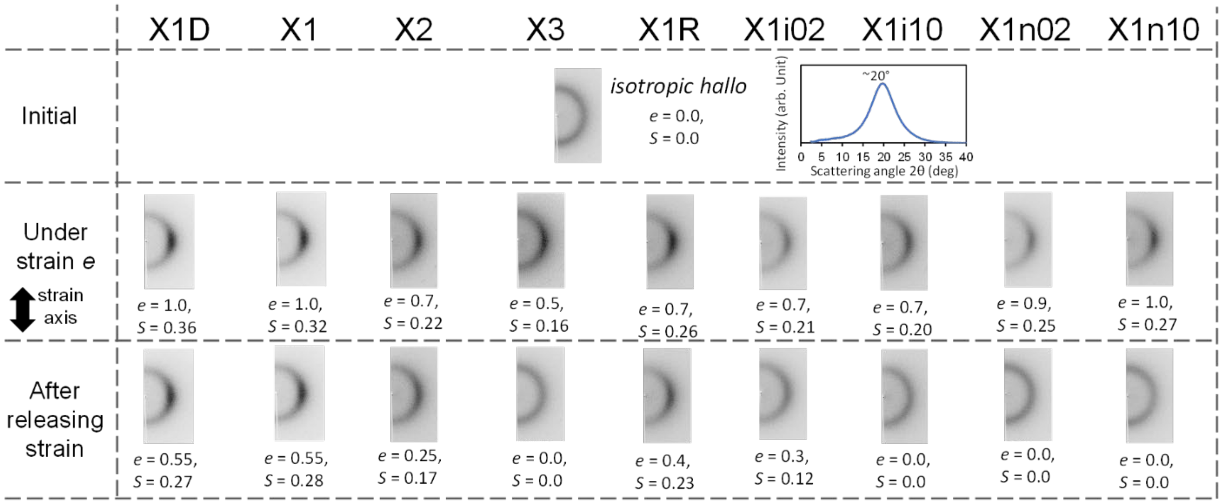

Supplementary Figure 9. WAXS of LCEs. The WAXS patterns are shown for LCEs under strain and after releasing strain. The strain and macroscopic orientational order parameter *S* are also shown. Before straining, all samples show isotropic hallo pattern at 2θ~20°. Note that present main-chain LCEs show no smectic/crystalline peak at small angle range independent of strain states, i.e., all LCEs studied here are in nematic phase.

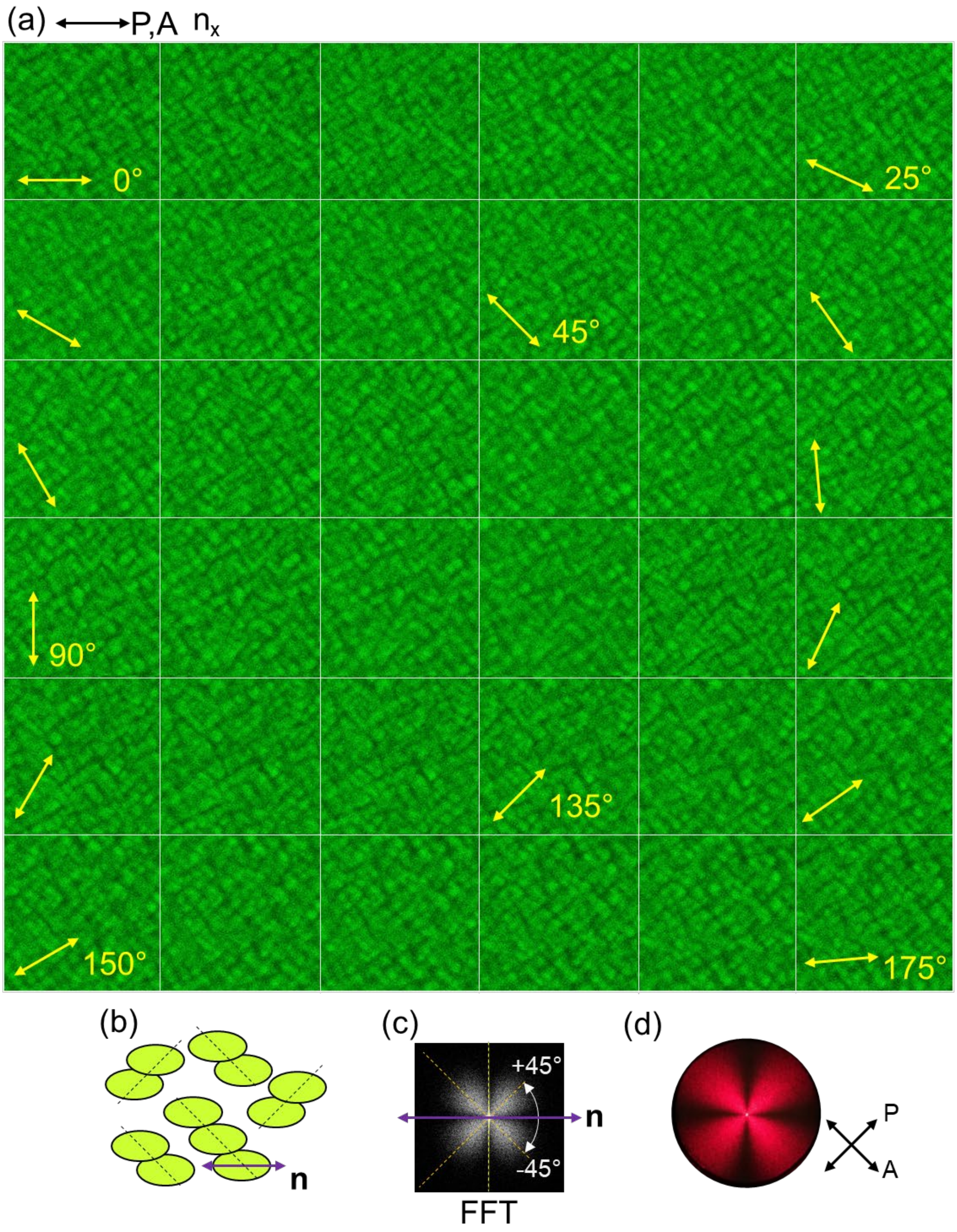

Supplementary Figure 10. PFOM images of polydomain structure of X1 at strain *e* = 0 on sample rotation. The polarisers are fixed in the *x* direction, while the sample was rotated at 5° intervals in the clockwise manner. $(15.3^2\ \mu m^2)$ The relative orientational correlation in the ±45° with respect to the director (in the *x* direction in images here) as shown in the schematic in (b) appears Independently of the rotation angle. This confirms that the polydomain structure state is macroscopically isotropic. The characteristic correlation of the polydomain state can be clearly recognised on the reciprocal space image (FFT) in (c). The qualitatively identical pattern is also obtained on DPLS pattern (d) taken on a thin sample.

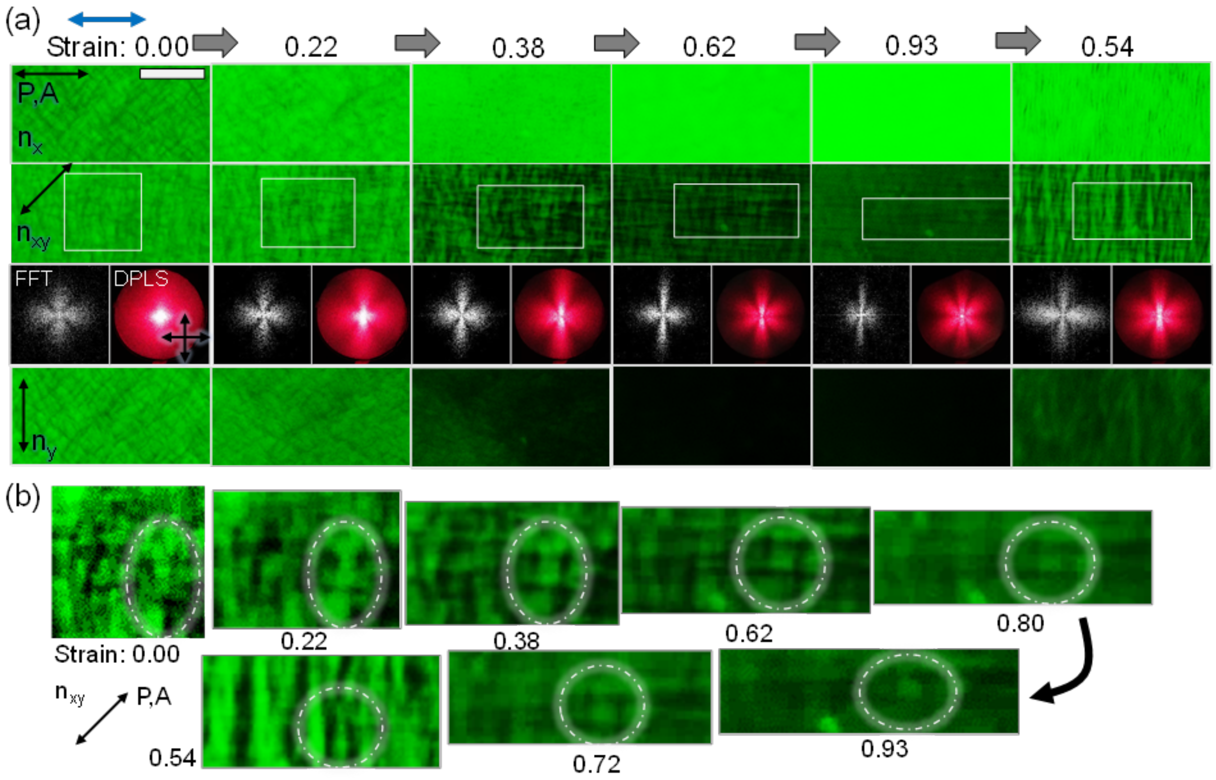

Supplementary Figure 11a-b. Nematic domain structures through strain cycle. (a) PFOM images with different polarisers angles on a stretching-releasing cycle on X1. Each fast Fourier transformed (FFT) on n_{xy} image and DPLS pattern with the crossed polarisers shown by the black arrows are also shown. (b) Magnified n_{xy} images at the location indicated by white rectangular parts on (a), which are initially 10^2 μm^2 in size, with the contrast enhanced. The dashed circles are guides for eyes to trace the domain transformation at each specific location. (Bar: 10 μm)

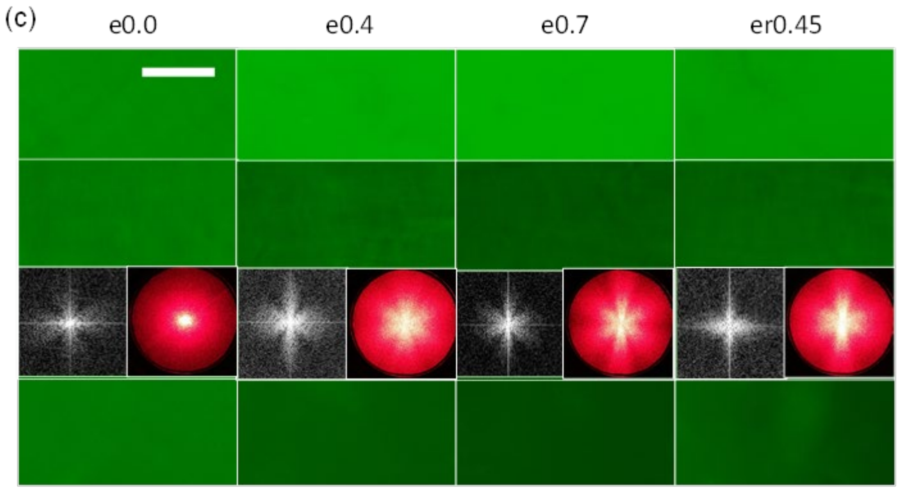

Supplementary Figure 11c. Nematic domain structures through strain cycle. (*continued*) (c) PFOM images with different polarisers angles on a stretching- releasing cycle on X1R. (Bar:

10 μm) Each fast Fourier transformed (FFT) on n_{xy} image and DPLS pattern with the crossed polarisers shown by the black arrows are also shown.

(d)

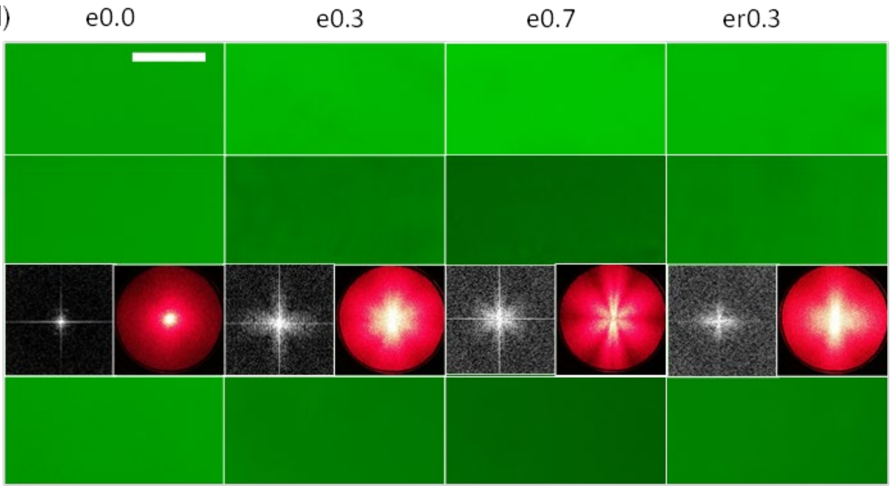

Supplementary Figure 11d. Nematic domain structures through strain cycle. (*continued*) (d) PFOM images with different polarisers angles on a stretching- releasing cycle on X1R. (Bar: 10 μm) Each fast Fourier transformed (FFT) on n_{xy} image and DPLS pattern with the crossed polarisers shown by the black arrows are also shown.

(e)

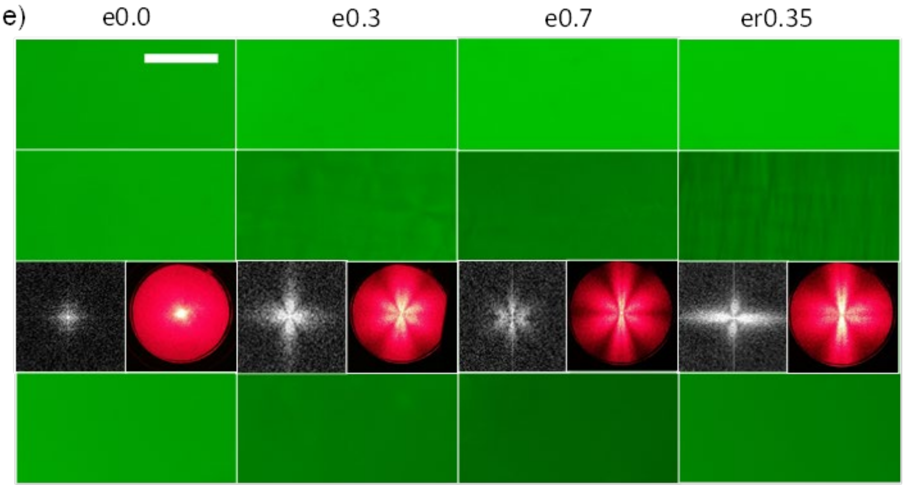

Supplementary Figure 11e. Nematic domain structures through strain cycle. (*continued*) (e) PFOM images with different polarisers angles on a stretching- releasing cycle on X1i02. (Bar: 10 μm) Each fast Fourier transformed (FFT) on n_{xy} image and DPLS pattern with the crossed polarisers shown by the black arrows are also shown.

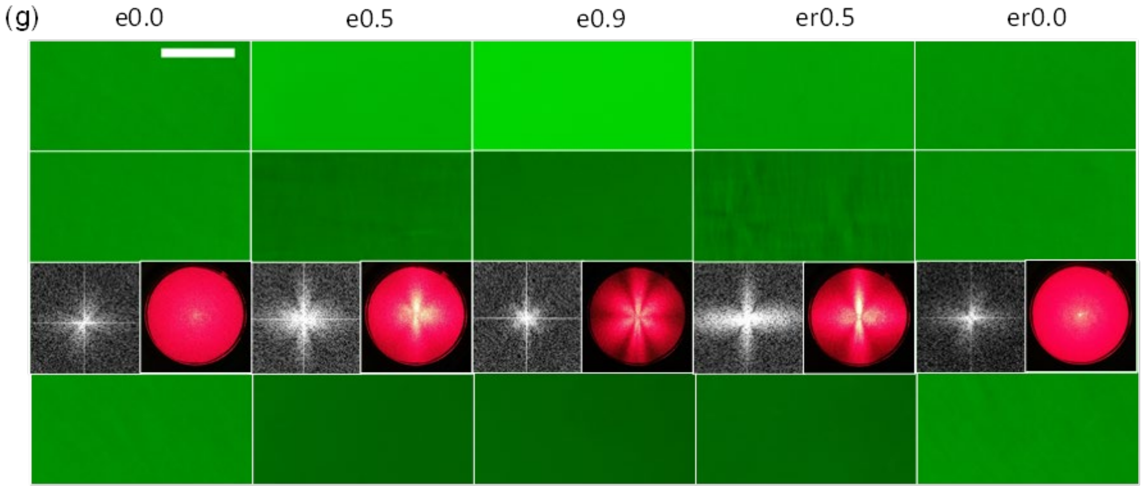

Supplementary Figure 11f. Nematic domain structures through strain cycle. (*continued*) (g) PFOM images with different polarisers angles on a stretching- releasing cycle on X1n02. (Bar: 10 μm) Each fast Fourier transformed (FFT) on n_{xy} image and DPLS pattern with the crossed polarisers shown by the black arrows are also shown.

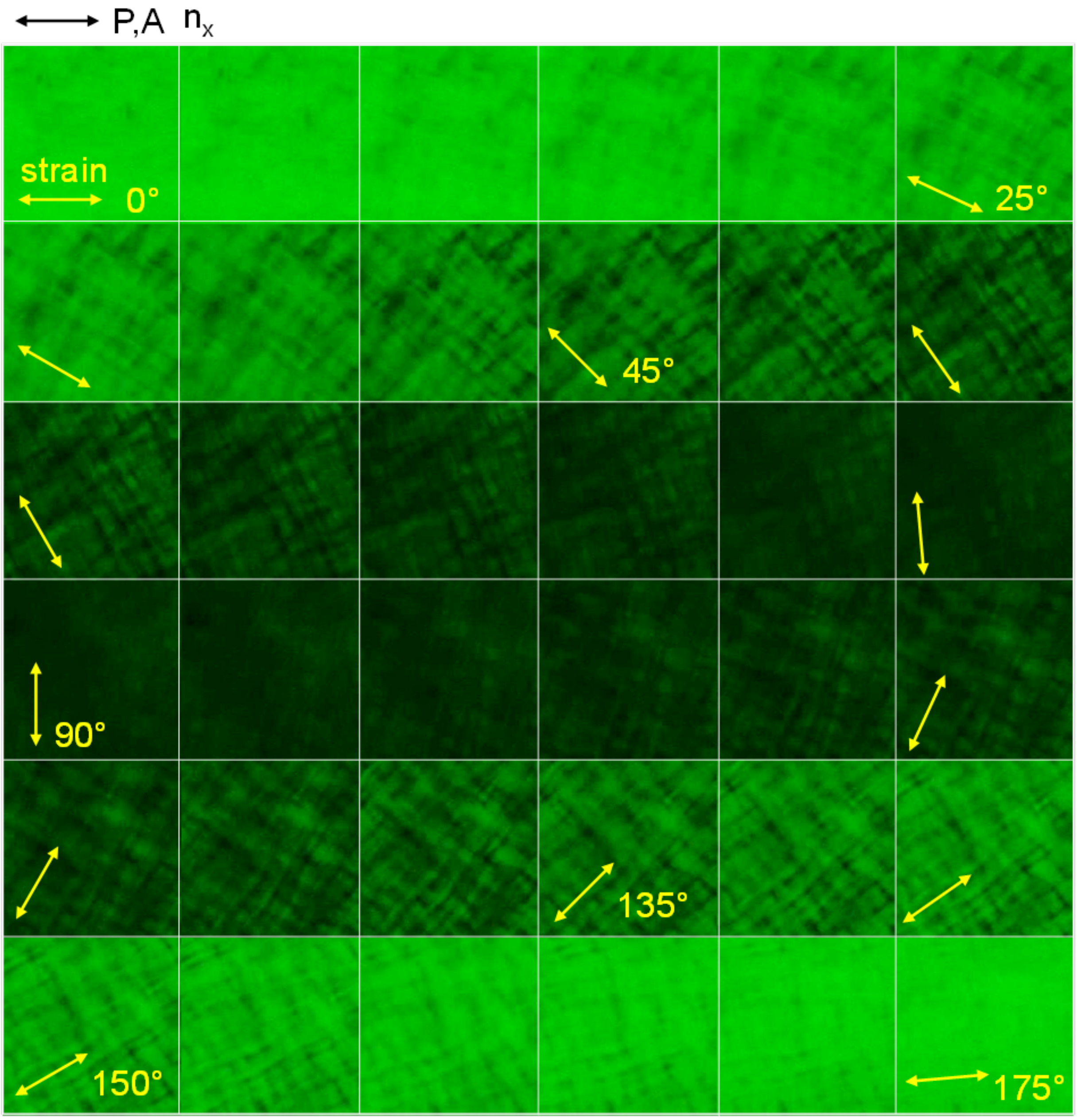

Supplementary Figure 12. PFOM images of typical lattice-like domain structure at strain of *e* = 0.6 on sample rotation (X1). The polarisers are fixed in the *x* direction, while the sample was rotated at 5° intervals in the clockwise manner. (15.3^2 μm^2)

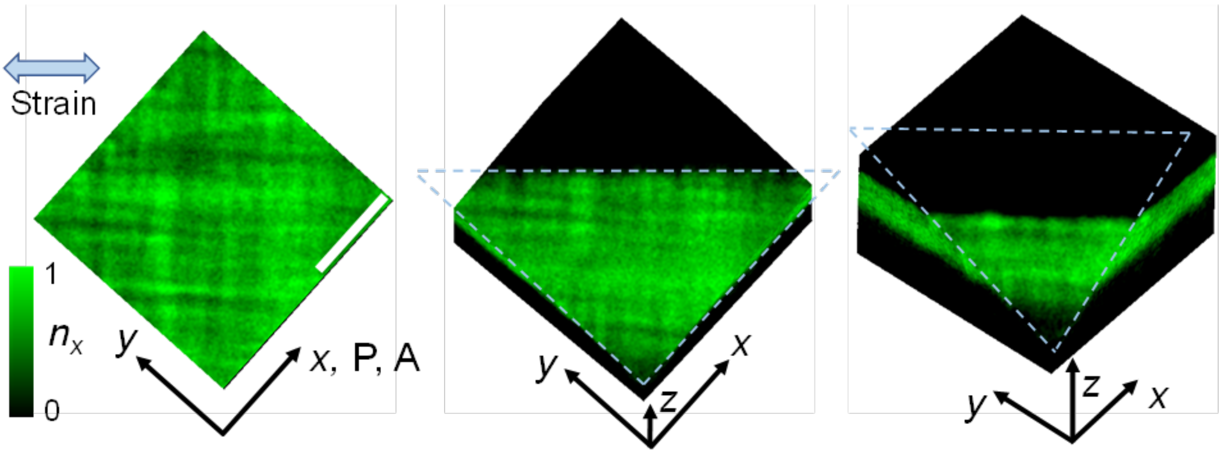

Supplementary Figure 13. Three-dimensional (3D) lattice-like domain structure at strain of *e* = 0.6 imaged by confocal PFOM (X1). The different cross-sections at the planes shown by dashed triangles show that the lattice-like structure spans three-dimensionally. (Bar 3 μm)

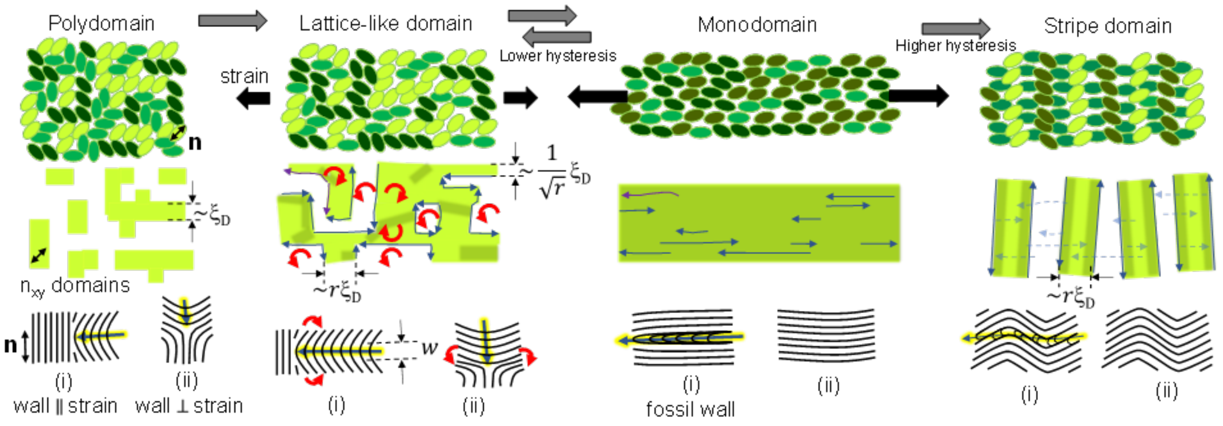

Supplementary Figure 14. Two-dimensional expression of the domain transformation scenario. The polydomain state (left) is macroscopically isotropic, showing the relative orientational correlation in the ±45° with respect to the director. When domains with **n** in the 45° from the horizontal axis are highlighted (n_{xy} domains shown in Fig 3 and Fig. S10), the rectangular shapes appear as shown in the middle-left illustration. Two key situations of the domain walls are shown on the bottom row; the domain wall is (i) parallel and (ii) perpendicular to the strain direction. As strain increases, each domain starts to rotate softly. If a plane of interest is chosen, which is the page plane shown here, there is two alternative rotational directions; clockwise (CW) or counterclockwise (CCW) as shown by red curved arrows. Although most of the directors "know" the direction of rotation that simply decreases the absolute angle with respect to the strain axis, the domains with director almost perpendicular to the strain axis have two choices. Since, for making a choice, it is energetically favourable to be triggered by the neighbouring domain walls rather than creating a new wall within the domain, the inherent domain walls in the polydomain will survive and grow as shown by blue arrows.

There are two distinct ways of the anisotropic growth shown as (i) and (ii), which run parallelly and perpendicularly to the strain axis. Although the inherent domain walls exist in any direction, these two are selected as the result of uniaxial stretching that breaks symmetry. Other walls would simply fade upon alignment. As the result, the rectangular lattice-like domain pattern should form, which has been roughly estimated from the DPLS pattern in the previous study by Clarke et al[2,3]. The characteristic lengths in two direction should change, which is indeed observed, depending on the relative length to the original one in the strain direction; as $r\xi_D$ and $\sim\frac{1}{\sqrt{r}}\xi_D$ in directions parallel and perpendicular to the strain axis, respectively, where r measures the anisotropy of the average chain shape spheroid (see also Fig. 4b,d) and ξ_D is the initial characteristic domain length ~1 μm. Upon further stretching, alignment proceeds toward the monodomain state and the domain walls type (i) would remain as fossil, which are assumed to be too narrow to be resolved (i.e., small *w*) and type (ii) can disappear. Note that due to the LC Frank elasticity penalising the sudden change in **n**, the domain walls in LCEs[4–6] should be not sharp as those in the crystals[7], e.g., twin boundaries.

The domain patterns after releasing strain depend on the degree of system's hysteresis. For example, X1n02 and 10 show almost reversible transformation (Supplementary Fig.10f and Fig. 3e) On X1, X1R, X2, X3, X1i02, and X1i10, the lattice-like pattern reappears with slight changes (Fig. S10a-f). On X1D, the clear stripe pattern, which has been known to be formed in the monodomain(∥)-monodomain(⊥) transition[5,8–10], forms (Fig. 3a,b, and Supplementary Figs. 13, 14), suggesting that X1D is the most hysteretic.

Note that, for simplicity, the two-dimensional expression is presented here. In actual systems, the macroscopic rotational symmetry with respect to the strain axis suggests that the present schematics can be applied to any plane including strain axis. 3D images of confocal PFOM (Supplementary Fig.14) and XRD patterns (Supplementary Fig.9), which show monotonical growth at peaks corresponding to the alignment in the strain direction without the split, also support this picture. This further suggests that the domain walls may be the lines in 3D situation, which is similar to the deformed situation known as the escaped directors into third dimension recognised as integer topological defects in ordinary liquid crystal systems[6,11].

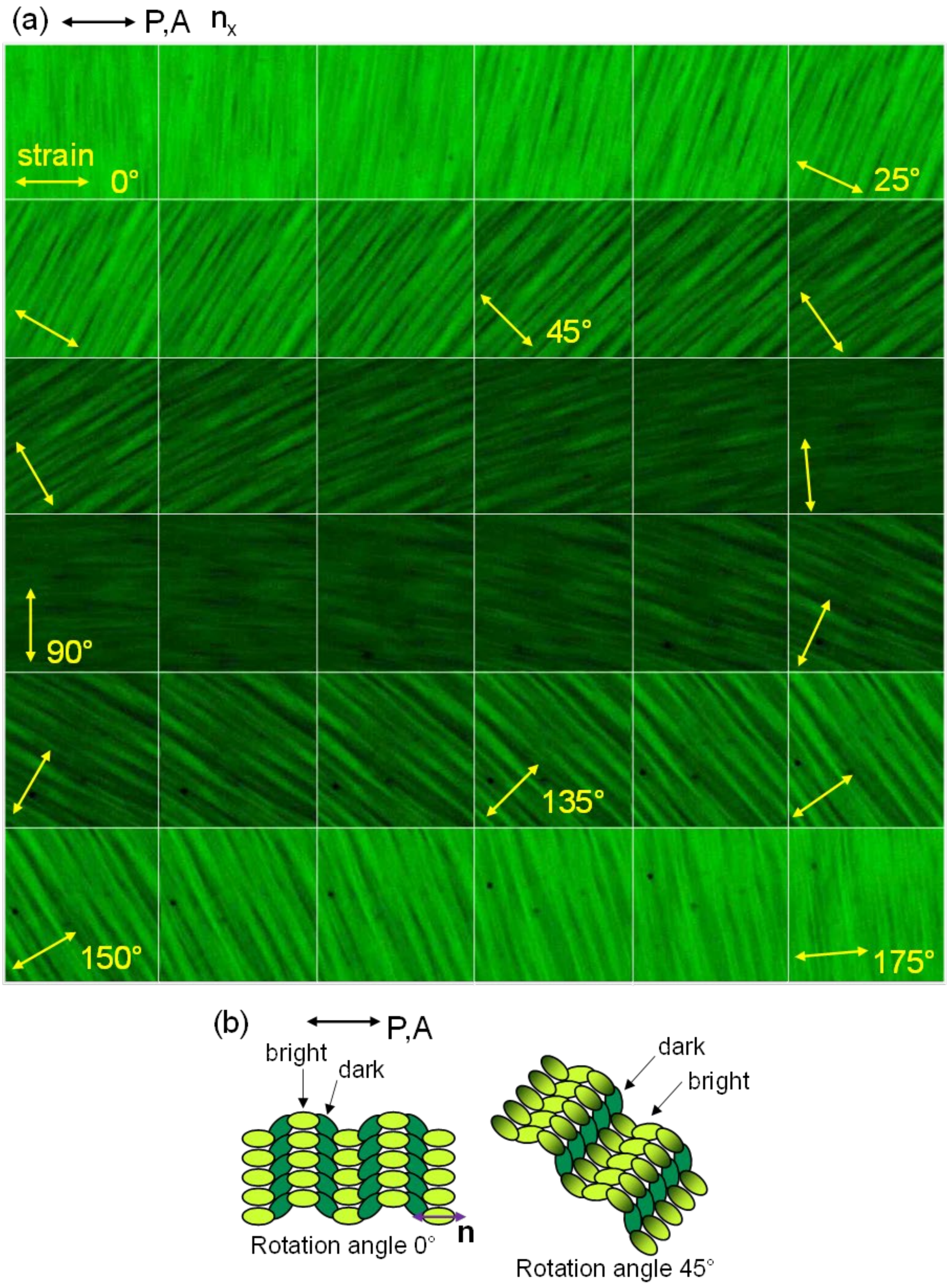

Supplementary Figure 15. PFOM images of stripe domain structure at strain of 0.6 after des-stretching on sample rotation (X1D). (a) The polarisers are fixed in the *x* direction, while the sample was rotated at 5° intervals in the clockwise manner. (15.3^2 μm^2) Note that the smaller periodicity of the stripes at the angle 0° than that at ~45°. This supports that the stripe pattern is the undulated/zigzag director pattern shown schematically in (b). This stripe pattern can be considered as the qualitatively the same as that reported for monodomain LCE samples, which is caused by the buckling instability of the monodomain state under compressive strain in their

initial alignment direction[5,8–10]. See Supplementary Movie 1 for the macroscopic glittering appearance.

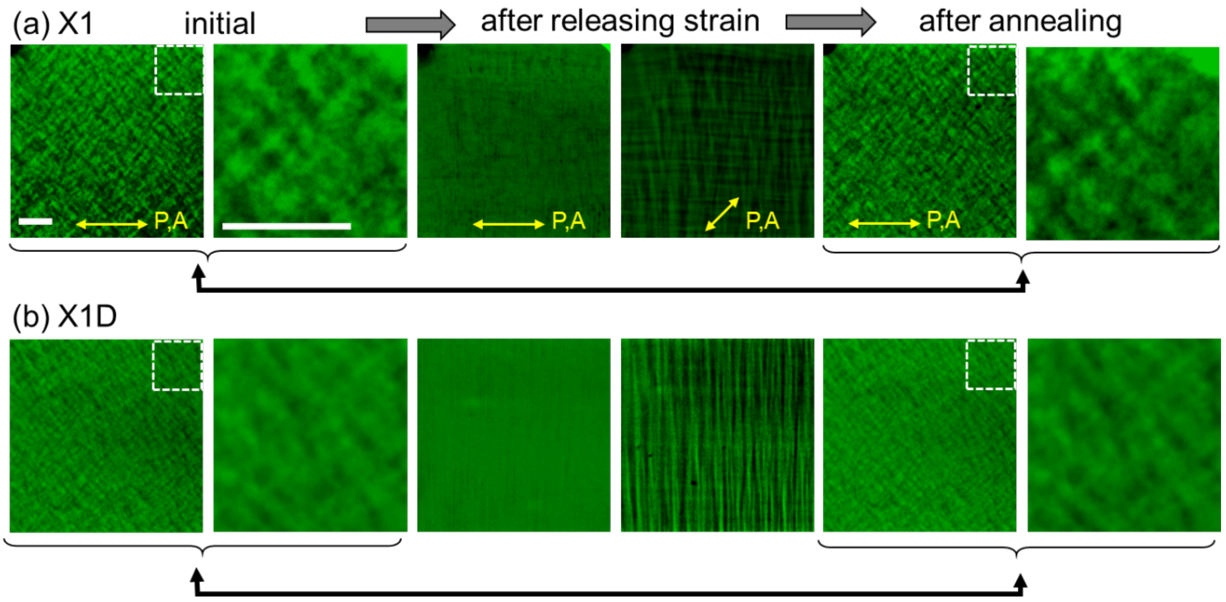

Supplementary Figure 16. PFOM images at 20 °C of initial and final samples after a stretching-releasing cycle followed by annealing at 80°C for 5 min at the same locations. The maximum applied strain was ~1.0. (a) X1 and (b) X1D show the almost total recovery of the initial polydomain structure, suggesting that the polydomain pattern is memorised in the network structure frozen by the chemical crosslinks. Moreover, the results suggest no noticeable permanent change in polymer network. For comparison, the magnified images, at the white squares in the larger scale images, are also shown. (Bar 5 μm)

Supplementary Note 1. Estimation of the energy potential barrier $\boldsymbol{G = G_{nem} + G_{el}}$ for the transition state upon hairpin glide. To simplify the transient state, the configurations shown in Fig. 4f are considered here. The energy states of neighbouring energy minima are assumed to be the same. Then, the energy increases due to the transient state correspond to G. The contributions from the nematic and elastic mean fields are separately evaluated as follows.

The increase of nematic mean field energy may be evaluated considering the anchoring-energy-like Meier-Saupe type energy[12,13], $F_{nem} = k_{\mathrm{B}}T\epsilon \int_{surface} P_2(\boldsymbol{n}\cdot\boldsymbol{t}[s])\mathrm{d}s$, where $\boldsymbol{n}$ is the fixed director of the surrounding mean nematic field, $\boldsymbol{t}$ is the unit vector normal to the molecular surface s that interacts with the nematic field, P_2 is the second order Legendre polynomial and the positive constant ϵ is the system-dependent nematic interaction parameter with unit of L^{-2}. ϵ is a function of the temperature via the nematic order parameter $Q(T)$ with $\frac{d\epsilon}{dQ} > 0$, and thus, should reflect all nematic interactions, e.g., dipole–dipole interaction and hydrogen bond. The value $\epsilon{\sim}5\ \mathrm{nm}^{-2}$, which has been reported for the low molecular weight nematic liquid crystals[12], is approximately adopted as the present nematic state of LCE. The molecular surfaces are approximated by the cylinders for rigid mesogenic part and other flexible spacer ones, with their cross-sectional diameters of the cylinders $d_m{\sim}0.7$ nm and $d_s{\sim}0.5$ nm, respectively, expected from the chemical formulae. (Note that the cross-sectional area of a long alkane chain is known as ~0.21 nm^2, ref[14]) Although other complicated configurations are possible, at least these cylinders must be perpendicular to the nematic mean field. The effective length of the rigid mesogenic part with phenyl moieties is also estimated as $L{\sim}1.8$ nm. Since they are on the polymer chain, the effective surface in the present description is the curved part alone. At the state before/after the transition, $\boldsymbol{n}\cdot\boldsymbol{t} = 0$. At the transition state, $\boldsymbol{n}_{hairpin}$ of the three cylinders shown in Fig. 4f become perpendicular to the surrounding nematic mean field, i.e., $\boldsymbol{t}$ become partially perpendicular to $\boldsymbol{n}$ as $\boldsymbol{n}\cdot\boldsymbol{t} = \cos\varphi$, where φ is the angle between $\boldsymbol{n}$ and $\boldsymbol{t}$. The total area Σ of interest may be the sum of curved area of the three cylinders as $\Sigma \approx$

$\pi(d_m + d_s)L{\sim}6.8\ \mathrm{nm}^2$. Then the energy barrier can be calculated as $G_{nem} = F_{nem-\mathrm{transition}} - F_{nem-\mathrm{before\cdot after}} = k_\mathrm{B}T\epsilon \int_0^{2\pi} [P_2(\cos\varphi) - P_2(0)] \frac{(d_m+d_s)}{2} L\mathrm{d}\varphi = \frac{3}{4} k_\mathrm{B}T\epsilon\Sigma{\sim}26k_\mathrm{B}T$. Even if the oversimplified cylinders for the molecular shapes and other approximations are considered, G may be larger than $k_\mathrm{B}T$ by one order of magnitude. Note that G_{nem} should rapidly decrease with increasing temperature toward T_NI and becomes zero at T_NI via $\epsilon[Q(T)]$.

The increase of elastic mean field energy may be roughly evaluated as $G_{el} \approx \mu\Delta v$, where μ is the effective elastic modulus in the order of ~0.1 MPa and Δv is the effective volume of deformation approximated as $\Delta v \approx \pi\left(\frac{{d_m}^2 + {d_s}^2}{4}\right) L{\sim}1\ \mathrm{nm}^3$. Then, $G_{el}{\sim}10^{-22}\ \mathrm{J}{\sim}0.025k_\mathrm{B}T$ at room temperature, which is much smaller than the above nematic effect and negligible here. For more accurate evaluation, the treatment by Argon[15] for the plastic deformation of glassy polymers may be useful.

The transition rate is then expressed as $f \exp\left(\frac{G}{k_\mathrm{B}T}\right)$, where f is the trial frequency typically in the THz range of molecular vibrations. For example, with $\frac{G}{k_\mathrm{B}T}{\sim}26$, $f \exp\left(\frac{G}{k_\mathrm{B}T}\right){\sim}5\ \mathrm{s}^{-1}$ for glide of single hairpin involving the π-rotation of the mesogenic part, which is already quite rare. Thus, if a pair of neighbouring transition events are coupled as in Fig. 4g, e.g., G is doubled, it is easily frozen by the exponential factor. This mechanical coupling of the neighbouring glide motions[16] through a common crosslink may exist everywhere in present LCE, further restricting them from occurring upon thermal activation only.

Note that the estimated activation energy $G{\sim}G_{nem}$ is also required to form a new hairpin on the strand, in addition to the chemical potential for the hairpin[16,17]. Thus, the event may also be very rare. For more accurate estimation of present situation, molecular dynamics simulations and/or statistical treatment of the polymer configurations would be required with the corresponding experimental characterisation of G, which remain as the future theoretical and experimental studies.

References in Supplementary Information

1. Biggins, J. S., Warner, M. & Bhattacharya, K. Supersoft elasticity in polydomain nematic elastomers. *Phys. Rev. Lett.* **103**, 1–4 (2009).
2. Clarke, S. M., Terentjev, E. M., Kundler, I. & Finkelmann, H. Texture evolution during the polydomain-monodomain transition in nematic elastomers. *Macromolecules* **9297**, 4862–4872 (1998).
3. Clarke, S. M., Nishikawa, E., Finkelmann, H. & Terentjev, E. M. Light-scattering study of random disorder in liquid crystalline elastomers. *Macromol. Chem. Phys.* **198**, 3485–3498 (1997).
4. Warner, M., Terentjev, E. M. *Liquid Crystal Elastomers*. (Oxford Univ. Press, 2007).
5. Verwey, G. C., Warner, M. & Terentjev, E. M. Elastic instability and stripe domains in liquid crystalline elastomers. *J. Phys. II* **6**, 1273–1290 (1996).
6. de Gennes, P. G. & Prost, J. *The Physics of Liquid Crystals*. (Oxford Univ. Press, 1993).
7. Salje, E. K. *Phase Transitions in Ferroelastic and Co-elastic Crystals*. (Cambridge University Press, 1993).
8. Kundler, I. & Finkelmann, H. Strain-induced director reorientation in nematic liquid single crystal elastomers. *Macromol. Rapid Commun.* **16**, 679–686 (1995).
9. Conti, S., DeSimone, A. & Dolzmann, G. Semisoft elasticity and director reorientation in stretched sheets of nematic elastomers. *Phys. Rev. E* **66**, 8 (2002).
10. Uchida, N. Soft and nonsoft structural transitions in disordered nematic networks. *Phys. Rev. E* **62**, 5119–5136 (2000).
11. Ohzono, T. *et al.* Uncovering different states of topological defects in schlieren textures of a nematic liquid crystal. *Sci. Rep.* **7**, 1–13 (2017).
12. Ferrarini, A., Moro, G. J., Nordio, P. L. & Luckhurst, G. R. A shape model for molecular ordering in nematics. *Mol. Phys.* **77**, 1–15 (1992).
13. Ohzono, T., Yatabe, T., Wang, C., Fukazawa, A. & Yamaguchi, S. Negative fluorescence anisotropy of phosphole oxide-based dyes in nematic liquid crystals. *Commun. Chem.* **1**, 52 (2018).
14. J N Israelachvili. *Intermolecular and Surface Forces*. *Intermolecular and Surface Forces* (Academic Press, 2011).
15. Argon, A. S. A theory for the low-temperature plastic deformation of glassy polymers. *Philos. Mag.* **28**, 839–865 (1973).
16. Adams, J. M. & Warner, M. Hairpin rubber elasticity. *Eur. Phys. J. E* **16**, 97–107 (2005).
17. de Gennes, P. G. Mechanical properties of nematic polymers. in *Polymer Liquid Crystals* (eds. Cifferi, H. von A., Krigbaum, W. R. & Meyer, R. B.) (Academic Press, 1982).
