# Supplementary material for: Internal constraints and arrested relaxation in main-chain nematic elastomers
Source: Nat Commun. 2021 Feb 4;12:787. doi: 10.1038/s41467-021-21036-3 (PMC7862651; doi:10.1038/s41467-021-21036-3)
Supplement: Supplementary file 2 — Description of additional supplementary information file [file 41467_2021_21036_MOESM2_ESM.pdf]

Supplementary Movie 1. Glittering of the X1D sample with residual strain after unloading from the strain of *e* ~1 under ambient room light. The glittering originates from the microscopic striped nematic domains (Figs. 3a,3b, and Supplementary Fig. 15) with the spatial length scale close to the wavelength of visible light. It is assumed that the regular structure with the periodic effective refractive index causes viewing-angle-dependent light scattering as in the general structural colouring. The appearance of the transparent isotropic state at elevated temperature are also shown as references.
